# Swine veterinarians' awareness, attitudes, and intention to recommend precision livestock farming technologies to clients

**DOI:** 10.3389/fvets.2026.1841102

**Published:** 2026-06-15

**Authors:** Babatope Akinyemi, Janice Siegford

**Affiliations:** Department of Animal Science, Michigan State University, East Lansing, MI, United States

**Keywords:** animal welfare, precision livestock farming, survey, swine veterinarians, technology adoption, theory of planned behavior

## Abstract

**Introduction:**

Precision Livestock Farming (PLF) technologies offer substantial promise for enhancing animal welfare, productivity, and disease detection in commercial swine production. Swine veterinarians hold a critical advisory role in bridging the gap between the technical evidence base for PLF and on-farm adoption decisions. Yet, the psychological and contextual factors that predict veterinarians' intention to recommend PLF to their clients remain poorly understood in the literature.

**Methods:**

Using survey data from a convenience sample of 61 U.S. swine veterinarians recruited across four professional meetings (July 2022–July 2023), we used a TPB-informed framework to model PLF recommendation intention and veterinarian-reported current client PLF use. Because perceived behavioral control was not directly measured, the analysis represents a partial rather than full operationalization of TPB. Five TPB-aligned constructs—PLF Awareness, Expected PLF Impact, PLF Cost Perception, PLF Subjective Norms, and PLF Help Wanted—underwent preliminary construct validation using exploratory factor analysis (EFA) with Kaiser–Meyer–Olkin (KMO) and Bartlett's sphericity diagnostics, supplemented by Cronbach's α and McDonald's ω (all α ≥ 0.70). Hierarchical binary logistic regression models were estimated for two outcomes: (A) veterinarian intention to recommend more PLF to clients, and (B) whether any of the veterinarian's clients currently use any PLF technology. Mann–Whitney *U*-tests examined construct differences between outcome groups; given the modest *n*, results are reported as exploratory and uncorrected *p*-values are flagged for caution against multiple testing.

**Results:**

PLF Help Wanted (*M* = 3.91) and Expected PLF Impact (*M* = 3.78) received the highest mean scores; PLF Cost Perception (*M* = 2.95) was near the scale midpoint, reflecting ambivalence about financial investment. Of 55 respondents who answered the recommendation question, 74.5% intended to recommend more PLF. Veterinarians intending to recommend PLF reported significantly higher Expected PLF Impact, PLF Cost Perception, PLF Subjective Norms, and PLF Help Wanted scores than those who did not recommend (all *p* < 0.05). The full logistic regression model for recommendation intention (Model A3) explained 51.0% of variance (Nagelkerke *R*^2^ = 0.510). Adding current client PLF use provided no incremental predictive value (Model A4 vs. A3: AIC 44.0 vs. 44.1; lower AIC = better fit; ΔAIC = 0.1 is negligible). For Outcome B (veterinarian-reported current client PLF use), PLF Awareness was the only psychological predictor reaching statistical significance under conventional maximum-likelihood standard errors (Model B2: *OR* = 3.07, 95% CI: 1.09–8.62, *p* = 0.034); however, this association was not robust to HC3 heteroscedasticity-consistent standard errors (*z* = 1.43, *p*_robust = 0.153) and is therefore presented as an exploratory association requiring confirmation in larger samples.

**Discussion:**

U.S. swine veterinarians in this convenience sample show heterogeneous engagement with PLF. Evaluative beliefs about PLF impact and cost-effectiveness, perceived norms, and recognition of client need for PLF emerge as candidate modifiable targets for extension and professional development. Cost perception was near the scale midpoint and warrants explicit attention in PLF outreach. As an exploratory, partially TPB-grounded study with limited statistical power, these findings should be interpreted as hypothesis-generating and require confirmation in larger, nationally representative samples.

## 1 Introduction

Pork is the most widely consumed meat globally, representing approximately 34% of total meat intake (1). Global pork consumption increased by roughly 77% between 1990 and 2022—from 63.5 to 113 million tons—and further increases are projected as population growth and rising incomes in middle-income countries continue to drive demand (1, 2). Against this backdrop, the swine industry faces a triple challenge: it must simultaneously scale output, reduce its environmental footprint, and meet rising societal expectations for animal welfare and food-safety transparency. Achieving all three goals requires tools that extend human monitoring capacity well beyond what is achievable through the stockperson's unaided observation, particularly as herd sizes grow and the stockperson-to-animal ratio decreases (3, 4).

Precision Livestock Farming (PLF) offers a promising toolset. Broadly defined as the application of process-engineering principles and automated sensor technologies to continuous, real-time monitoring of individual livestock (5, 6), PLF encompasses cameras, microphones, accelerometers, infrared thermography units, flow meters, and RFID systems—each translating raw biological signals into actionable welfare and productivity indicators (4, 7, 8). In swine production specifically, validated PLF tools can detect early signs of lameness, respiratory disease, thermal stress, tail biting, body condition deterioration, and reproductive problems with a speed and consistency that human observation cannot match (3, 4, 7).

Despite this technological momentum, PLF adoption on commercial U.S. swine farms remains uneven, and research on the human dimensions of that adoption gap is strikingly limited (9). Collins and Smith (3) identify four systemic barriers to PLF uptake: difficulty integrating multiple sensor platforms without in-house analytical expertise; insufficient cross-system validation; data-sharing and ownership complications; and the high investment risk associated with emerging technologies. These structural barriers coexist with important psychological barriers that have received far less empirical attention in the U.S. context.

Swine veterinarians are a strategically critical yet underexplored node in the PLF adoption chain. As clinically trained advisors who visit farms regularly, interpret herd health and production data, and make concrete recommendations to producer clients, they are uniquely positioned to bridge PLF's technical evidence base and on-farm adoption decisions (7, 10). Qualitative evidence from European contexts suggests that pig veterinarians view PLF as a valuable advisory tool but also navigate financial dependencies and competing stakeholder interests (10). Within the U.S. swine industry, stakeholder perceptions of PLF have been explored through qualitative and *Q*-methodology approaches (11, 12), revealing divergent views on PLF's ability to address core welfare and production challenges; however, the veterinarian-specific perspective remains unquantified. No quantitative study has measured which psychological and contextual factors most strongly predict a U.S. swine veterinarian's intention to recommend PLF to clients—or whether that intention varies by experience, specialty, or existing client adoption behavior.

Available evidence supports the assumption that veterinarians shape on-farm investment and management decisions. Surveys of U.S. food-animal producers consistently identify the herd veterinarian as among the most trusted and most consulted sources of advice on health, welfare, and production-related interventions (13–15). In swine specifically, regular veterinary visits and herd-health programs are credited with driving uptake of vaccination protocols, disease-monitoring tools, and biosecurity practices (16, 17). Producer adoption of PLF has been associated with information from advisors and peer farmers, perceived ease of integration with existing management, and clear cost–benefit signals (9, 18). These findings provide the empirical foundation for examining swine veterinarians as influential—rather than peripheral—actors in the PLF adoption chain.

The present study directly addresses this gap by examining three exploratory research questions in a convenience sample of U.S. swine veterinarians. First, which psychological constructs—evaluative beliefs about PLF impact and cost, perceived subjective norms, recognition of client need for PLF assistance, and awareness of available PLF technologies—are most strongly associated with veterinarians' intention to recommend more PLF to their clients? Second, do the same constructs distinguish veterinarians whose clients currently use any PLF technology, as reported by the veterinarian, from those whose clients do not? Third, to what extent do sociodemographic and practice characteristics (gender, age, years of professional experience, practice specialty, and geographic location) account for variation in these two outcomes? Addressing these questions provides, to our knowledge, the first quantitative, TPB-informed evidence base on the psychological and contextual correlates of PLF advisory engagement among U.S. swine veterinarians.

## 2 Materials and methods

### 2.1 Ethics statement

This study was evaluated by the Michigan State University Institutional Review Board and determined to be exempt from the requirement for closer ethical scrutiny. All participants confirmed they were 18 years of age or older and indicated their consent before participating in the survey.

### 2.2 Study design and participant recruitment

A cross-sectional survey design was employed using a structured, self-administered paper questionnaire. The target population was practicing U.S. swine veterinarians. Participants were recruited through extension members of the IDEAS project, who have established relationships with swine veterinarians across the United States. Across four swine veterinarian professional meetings held from July 2022 to July 2023, the questionnaire was administered in a standardized format. Each session began with a brief presentation introducing the study topic and survey structure. Participants then completed an informed consent form and an initial PLF awareness question. A second presentation provided a standardized definition of PLF alongside examples of commercially available PLF tools, ensuring all respondents were exposed to an identical operational definition before proceeding. Sociodemographic characteristics (gender, age category, years of professional experience, practice specialty, and office geographical location) were placed at the end of the questionnaire to minimize priming effects. A total of 61 swine veterinarians completed the survey; the recruitment was therefore a convenience sample drawn from professional meeting attendees rather than a probability sample of U.S. swine veterinarians. A copy of the full questionnaire is provided as Supplementary material.

### 2.3 Theoretical framework

This study is best characterized as TPB-informed (i.e., partially TPB-grounded) rather than as a complete operationalization of the Theory of Planned Behavior. The study was guided by the Theory of Planned Behavior (TPB) (19), which has been widely applied in agricultural technology adoption research (20, 21). The TPB posits that behavioral intention is jointly determined by: (1) attitude toward the behavior, operationalized here as evaluative beliefs about PLF adoption; and (2) subjective norms, defined as perceived social pressure from relevant referents to engage in the behavior. A third standard TPB component, perceived behavioral control, was not directly measured in this study. The target behavior here is the veterinarian's advisory recommendation rather than actual farm-level technology installation, and our original reasoning was that the recommendation behavior is comparatively less constrained by physical or financial barriers at the individual veterinarian level. We acknowledge, however, that PBC and self-efficacy are important determinants of advisory behavior (e.g., the veterinarian's confidence in evaluating PLF technologies, communicating cost–benefit tradeoffs, or responding to producer resistance). Their omission is therefore recognized as a theoretical limitation of the present design (see Section 4.7), and the analysis is presented as TPB-informed rather than as a full TPB framework. PLF technology awareness was additionally included as a prerequisite cognitive condition, consistent with Rogers' (22) diffusion of innovations framework, which stipulates that awareness-knowledge must precede the formation of evaluative attitudes toward an innovation.

We further acknowledged that PBC in TPB contexts encompasses not only physical access but also self-efficacy—the veterinarian's confidence in evaluating PLF technologies, communicating cost-benefit tradeoffs to producer clients, and navigating producer resistance. These self-efficacy dimensions are documented as meaningful barriers in comparable advisory adoption contexts (18, 20). Accordingly, the present study is best described as TPB-informed or partially TPB-grounded, rather than as a complete test of the full TPB framework. Future studies should incorporate validated PBC items to complete the TPB causal model in this population.

### 2.4 Questionnaire design

A structured, self-administered questionnaire was developed for the study. Section 1 assessed awareness of nine specific PLF technologies using a five-point Likert-type scale (1 = totally unaware to 5 = totally aware): microphone-based cough detection (23), camera-based pig weight estimation (24, 25), vibration sensors for piglet crushing prevention (26, 27), pressure-plate lameness detection (28), individual water intake monitoring via RFID (29), electronic weighing scales (4), RFID-based individual pig identification (20, 30), automated heat detection (31), and Electronic Sow Feeders (4).

Section 2 contained 11 attitude items organized across two conceptual sub-scales: eight items assessing Expected PLF Impact on pig welfare, farm profitability, and the veterinarian's own work experience; and three items assessing PLF Cost Perception (installation, operation, and maintenance costs). Section 3 assessed PLF Subjective Norms through six items evaluating perceived professional and social pressure to recommend PLF adoption to clients. Section 4 assessed veterinarians' perceived need for PLF-based assistance across nine production problem areas. Two binary outcome items captured whether any of the veterinarian's clients currently use PLF (yes/no) and whether the veterinarian intended to recommend more PLF to clients in the future (yes/no). Construct definitions and item wording are summarized in Supplementary Table S1, and a copy of the full instrument is provided in Supplementary material to support transparency and replication.

### 2.5 Construct development and scale reliability

Five constructs were operationalized: PLF Awareness (nine items), Expected PLF Impact (eight items), PLF Cost Perception (three items), PLF Subjective Norms (six items), and PLF Help Wanted (nine items). All Likert-type ordinal items were aggregated as arithmetic item means to form composite construct scores (32, 33).

Internal consistency was assessed using Cronbach's α for all five constructs prior to further analysis, following standard psychometric practice (34). As an additional reliability check, omega (ω) coefficients were computed using the psych package (35) to address known limitations of α under conditions of heterogeneous factor loadings (36, 37). A retention threshold of α ≥ 0.70 was adopted (38). Numerical reliability values are reported in the Results (Section 3.2, Table 1), in line with standard reporting practice and the reviewers' recommendation. Given the modest sample, we describe these procedures throughout as preliminary construct validation rather than full psychometric validation, recognizing that broader replication is required before strong claims of construct validity can be made (39, 40).

**Table 1:** Construct reliability and descriptive statistics.

Construct	Items	α	Interpretation	Mean	*SD*
PLF Awareness	9	0.796	Acceptable	3.50	0.83
Expected PLF Impact	8	0.893	Good	3.78	0.70
PLF Cost Perception	3	0.896	Good	2.95	1.08
PLF Subjective Norms	6	0.884	Good	3.11	0.95
PLF Help Wanted	9	0.837	Good	3.91	0.70

α, Cronbach's alpha; SD, standard deviation.

All constructs scored on a 1–5 Likert-type scale.

### 2.6 Analytical strategy

#### 2.6.1 Exploratory factor analysis

Exploratory Factor Analysis (EFA) was selected over confirmatory factor analysis (CFA) because the constructs were newly assembled for the swine veterinary advisory context and prior model specifications were not available; in this exploratory framing, EFA is the appropriate first step before later confirmatory testing (40, 41). EFA was conducted for each of the five construct blocks using minimum residual (OLS) estimation with oblique (oblimin) rotation, consistent with the expectation of correlated factors within constructs (42). Sampling adequacy and suitability for factor analysis were assessed using the Kaiser–Meyer–Olkin (KMO) measure and Bartlett's test of sphericity (43, 44); KMO ≥ 0.60 and a significant Bartlett's test were treated as supporting prerequisites for EFA. The number of factors to retain was determined by parallel analysis based on 100 iterations (45), which is widely regarded as the most accurate factor retention criterion. Item-level loadings were inspected, and items with primary loadings below 0.32 or cross-loadings above 0.32 on a secondary factor were candidates for removal; the 0.32 cut-off corresponds to approximately 10% shared variance and is the conventional minimum salience threshold for interpretation (46, 47). All EFA were conducted using the psych package (35) in *R* version 4.3. Reliability benchmarks followed Kline (38): α ≥ 0.70 was the minimum threshold for construct retention. Where parallel analysis suggested multidimensionality (PLF Subjective Norms; PLF Help Wanted), composite construct scores were retained as the primary unit of analysis to keep the regression models tractable for the available sample size, and subscale-based sensitivity analyses were conducted as a robustness check (results reported in Section 3.9 and the Supplementary material). Subscale-based sensitivity regressions are reported in Supplementary Tables S9, S10.

#### 2.6.2 Descriptive statistics and bivariate analyses

Descriptive statistics (mean, standard deviation) were computed for all retained construct scores. Given the ordinal item origins and moderately non-normal distributions of construct scores, Spearman rank correlations were used to quantify bivariate associations among constructs (48). Differences in construct scores between veterinarians who intended vs. did not intend to recommend PLF, and between those whose clients currently used vs. did not use PLF, were tested using the Mann–Whitney *U*-test (49). Associations between categorical demographic variables and binary outcomes were examined with Pearson chi-square tests, supplemented by Monte Carlo simulation (*n* = 2,000 replicates) when expected cell counts were small.

#### 2.6.3 Binary logistic regression

Two binary logistic regression analyses were performed to model: (A) the veterinarian's intention to recommend more PLF technologies (yes/no); and (B) whether any of the veterinarian's clients currently use any PLF technology (yes/no), as reported by the veterinarian. For each outcome, a hierarchical modeling strategy was adopted (50). Three nested models were estimated for each outcome and labeled accordingly: B1 (demographics only), B2 (constructs only), and B3 (full model with both blocks) for Outcome B, in parallel with A1, A2, and A3 for Outcome A. Model A1 included only sociodemographic predictors: years of experience (continuous midpoint-scored), gender (male = 1), and general-practitioner specialty (yes = 1). Practice location indicators were excluded due to near-perfect separation (51)—a condition in which one or more covariate values almost perfectly predict the outcome category, causing maximum-likelihood odds ratios and standard errors to inflate without bound—in the small sample, which produced unreliable odds ratios. Model A2 entered only the five TPB-aligned construct scores. Model A3 (full model) combined constructs and demographics. For Outcome A, Model A4 additionally included whether clients currently use PLF, testing whether observed adoption experience independently predicts recommendation intention. Regression was fitted on a fully complete-case analytical sample (*n* = 39) obtained by listwise deletion. Odds ratios (*OR*) and 95% confidence intervals (CI) are reported.

Given the modest events-per-variable ratio in Model A3 and residual concerns about separation in small samples, Firth penalized maximum-likelihood logistic regression (52, 53) was estimated as a sensitivity analysis. Firth penalization was chosen specifically because it (i) reduces the small-sample bias inherent in standard maximum-likelihood logistic estimates and (ii) returns finite, well-behaved parameter estimates even when one or more covariate patterns produce quasi-separation—conditions that apply directly to the present sample. Estimation was performed using the logistf package with profile-likelihood confidence intervals (54) (results in Supplementary Tables S5, S6). Pattern and proportion of missingness across all variables are summarized in Supplementary Table S2; in addition to mean imputation, multiple imputation by chained equations (*m* = 20) was implemented using the mice package (55) as a more principled missing-data sensitivity analysis (pooled results in Supplementary Tables S7, S8). Recognizing the modest sample size and the presence of multiple correlated tests, all multivariable models are described as exploratory and uncorrected *p*-values are presented; Benjamini–Hochberg (BH) false-discovery-rate adjustments were also computed for the bivariate Mann–Whitney comparisons in Table 2 (56) (adjusted *q*-values in Supplementary Table S11).

**Table 2:** Mann–Whitney *U*-tests.

Construct	Intention to recommend PLF			Client usage of PLF		
	*W*	*p*-value	Sig.	*W*	*p*-value	Sig.
PLF awareness	372.5	0.100		341.5	0.201	
Expected PLF impact	476.5	<0.001	^*^	306.0	0.593	
PLF cost perception	486.0	<0.001	^*^	285.0	0.925	
PLF subjective norms	379.5	0.014	^*^	273.0	0.891	
PLF help wanted	386.0	0.016	^*^	346.5	0.166	

^*^p < 0.05 (two-tailed, uncorrected).

W, Mann–Whitney U statistic.

Model fit was evaluated using the Akaike Information Criterion (AIC) (57), McFadden's pseudo-*R*^2^, and Nagelkerke's *R*^2^ (58). Lower AIC indicates better relative fit, with ΔAIC <2 typically interpreted as no meaningful difference between competing models, ΔAIC of 4–7 as moderate evidence of difference, and ΔAIC >10 as strong evidence (59). Higher McFadden's and Nagelkerke's pseudo-*R*^2^ values reflect greater explained variance, but, unlike AIC, they generally increase as predictors are added and do not by themselves indicate that an added predictor materially improves model performance (60). Multicollinearity was assessed via the Variance Inflation Factor (VIF; 39); VIF <5 was interpreted as acceptable. Influential observations were identified using Cook's distance (61) (threshold: 4/*n*). Heteroscedasticity-consistent (HC3) robust standard errors were estimated as a robustness check (62, 63). A mean-imputation sensitivity analysis (*n* = 47) was conducted to evaluate potential bias from listwise deletion. Model separation was assessed using Hosmer–Lemeshow goodness-of-fit tests and VIF inspection; for the Hosmer–Lemeshow test, *p* > 0.05 indicates adequate calibration (64). All analyses were conducted in *R* version 4.3 using the psych, lmtest, sandwich, car, and ggplot2 packages (35, 65–67), supplemented by logistf for Firth penalized regression (54) and mice for multiple imputation (55). A two-tailed significance threshold of α = 0.05 was adopted throughout.

Supplementary Figures S1–S4 present item-level EFA factor loading plots for each construct block; Supplementary Figure S5 shows construct score distributions; Supplementary Figure S6 is the Spearman inter-construct correlation matrix; Supplementary Figure S7 illustrates construct score distributions by outcome group;Supplementary Figures S8, S9 are forest plots of Models A3 and B3; Supplementary Figures S10, S11 display Cook's distance diagnostic plots for Models A3 and B3 respectively.

## 3 Results

### 3.1 Participant characteristics

The sociodemographic characteristics of the 61 participating U.S. swine veterinarians are summarized in Table 3. The largest gender group was male (44.3%), followed by female (39.3%), and 3.3% preferring not to disclose. The largest proportion of respondents practiced in Iowa (34.4%), followed by North Carolina (24.6%) and other states (27.9%). Most identified as general practitioners (70.5%). Years of experience were broadly distributed: the most common categories were ≤ 5 years (21.3%), ≥31 years (18.0%), and 16–20 years (18.0%), reflecting a sample that spanned both early-career and highly experienced practitioners.

**Table 3:** Sociodemographic characteristics of survey respondents (*N* = 61).

Variable	Frequency	Percent (%)
Gender		
Male	27	44.3
Female	24	39.3
Prefer not to disclose	2	3.3
Practice location		
Iowa	21	34.4
North Carolina	15	24.6
Other states	17	27.9
Specialty		
General practitioner	43	70.5
Other specialty	18	29.5
Years of experience		
≤ 5 years	13	21.3
6–10 years	7	11.5
11–15 years	5	8.2
16–20 years	11	18.0
21–25 years	6	9.8
26–30 years	8	13.1
≥31 years	11	18.0

Eight respondents did not respond to gender and practice location questions.

### 3.2 Construct reliability

All five constructs demonstrated acceptable to good internal consistency (Table 1): PLF Cost Perception (α = 0.896) and Expected PLF Impact (α = 0.893) achieved the highest values, followed by PLF Subjective Norms (α = 0.884), PLF Help Wanted (α = 0.837), and PLF Awareness (α = 0.796), all exceeding the α ≥ 0.70 retention threshold (38). Omega (ω) coefficients computed for each construct were commensurate with α values (range: 0.81–0.91), supporting the robustness of scale reliability estimates. Sample sizes varied modestly across constructs because of item-level missingness: PLF Awareness, Expected PLF Impact, and PLF Cost Perception were estimable for *n* = 61 respondents, while PLF Subjective Norms and PLF Help Wanted were estimable for *n* = 59 and *n* = 60, respectively (full missingness pattern reported in Supplementary Table S2).

### 3.3 Exploratory factor analysis (EFA)

EFA results are summarized in Table 4. PLF Awareness yielded a single dominant factor accounting for 32.2% of variance, with all nine items loading positively (range: 0.46–0.75; see Supplementary Figure S1). Expected PLF Impact supported a single-factor solution explaining 52.4% of variance (item loadings: 0.56–0.84; see Supplementary Figure S2). PLF Cost Perception was strongly unidimensional, with a single factor explaining 76.7% of variance (operation cost item loading: 0.99). PLF Subjective Norms divided into two sub-dimensions—informal/commercial channels (social media, client, and industry representative pressure) and formal professional channels (collegial and association pressure)—jointly explaining 73.5% of variance (see Supplementary Figure S3). PLF Help Wanted similarly split into sow-level (farrowing and reproductive) and group-level (growing-finishing) problem domains, explaining 49.0% of cumulative variance (Supplementary Figure S4).

**Table 4:** Factors retained and cumulative variance explained.

Construct	No. of factors retained	Cumulative variance explained (%)
PLF awareness	1	32.2
Expected PLF impact	1	52.4
PLF cost perception	1	76.7
PLF subjective norms	2	73.5
PLF help wanted	2	49.0

### 3.4 Construct scores and inter-construct correlations

PLF Help Wanted returned the highest mean score (*M* = 3.91, *SD* = 0.70), indicating broad veterinarian perception that clients welcome PLF-based assistance. Expected PLF Impact (*M* = 3.78, *SD* = 0.70) and PLF Awareness (*M* = 3.50, *SD* = 0.83) were above the scale midpoint, indicating generally positive evaluative attitudes and moderate-to-high technology awareness. PLF Cost Perception (*M* = 2.95, *SD* = 1.08) was near the midpoint, reflecting genuine ambivalence about financial viability. PLF Subjective Norms (*M* = 3.11, *SD* = 0.95) were slightly above the midpoint. Distributions of all construct scores are shown in Supplementary Figure S5.

Spearman correlations among constructs are presented in Supplementary Figure S6. The strongest association was between Expected PLF Impact and PLF Cost Perception (ρ = 0.71, *p* < 0.001), indicating that veterinarians who anticipated greater positive PLF impacts also perceived the associated financial investment as more justifiable. PLF Subjective Norms correlated positively with PLF Help Wanted (ρ = 0.33, *p* = 0.010) and PLF Cost Perception (ρ = 0.33, *p* = 0.013). PLF Awareness correlated positively with PLF Subjective Norms (ρ = 0.30, *p* = 0.021). These correlation patterns are directionally consistent with the TPB's predicted interrelations among attitude, subjective norms, and behavioral cognitions.

### 3.5 PLF technology awareness by technology type

Veterinarians' awareness of microphone-based cough detection, camera-based pig weight estimation, vibration sensors for piglet crushing prevention, pressure-plate lameness detection, individual water intake monitoring, electronic weighing scales, RFID-based individual pig identification, automated heat detection, and Electronic Sow Feeders were assessed. RFID-based individual pig identification was the most recognized (*M* = 4.69, *SD* = 0.67), with 39.3% of respondents reporting being ‘Totally Aware'. Electronic Sow Feeders ranked second (*M* = 4.51, *SD* = 1.09). Weighing scales (*M* = 3.75, *SD* = 1.41) and microphone-based cough detection (*M* = 3.70, *SD* = 1.39) occupied a middle tier. Camera-based weight technologies, while commercially emerging, were not yet widely recognized (68). Pressure-plate lameness detection (SowSIS; *M* = 2.62, *SD* = 1.49) was the least recognized technology overall, with 24.6% of respondents reporting being ‘Totally Unaware', and individual water intake monitoring received similarly modest awareness scores (*M* = 2.80).

### 3.6 Veterinarian-reported client PLF use and recommendation intention

Of 48 respondents who answered the client adoption question, 58.3% reported that at least some of their clients currently use at least one PLF technology. Of 55 respondents answering the recommendation intention item, 74.5% stated they intend to recommend more PLF technologies in the future. No significant association was found between recommendation intention and years of professional experience (Kruskal–Wallis H (6) = 3.26, *p* = 0.813). A chi-square test suggested that veterinarian-reported client PLF use varied by practice location (χ^2^ = 13.79, *p* = 0.003); however, location indicators were excluded from regression models due to near-perfect separation and are reported here for descriptive purposes only.

### 3.7 Logistic regression

#### 3.7.1 Predictors of recommendation intention (outcome A)

Four hierarchical binary logistic regression models were estimated for Outcome A (Table 5). The demographics-only baseline (Model A1) achieved McFadden's *R*^2^ = 0.117, Nagelkerke *R*^2^ = 0.180 (AIC = 45.2). General Practitioner specialty was the only significant predictor in this baseline model (*OR* = 6.944, *p* = 0.034). The constructs-only model (Model A2) yielded McFadden *R*^2^ = 0.271, Nagelkerke *R*^2^ = 0.385 (AIC = 42.7). The full model (Model A3; AIC = 44.1, McFadden *R*^2^ = 0.380, Nagelkerke *R*^2^ = 0.510) provided the most comprehensive characterization of recommendation intention. Adding current client PLF use as a predictor (Model A4; AIC = 44.0, McFadden *R*^2^ = 0.430, Nagelkerke *R*^2^ = 0.562) produced no meaningful incremental improvement, indicating that working with clients who currently use PLF does not independently predict recommendation intention once psychological constructs and demographics are accounted for. Three models were estimated for Outcome B. The demographics-only model (Model B1) achieved McFadden *R*^2^ = 0.121, Nagelkerke *R*^2^ = 0.204 (AIC = 54.4); no single demographic predictor reached conventional significance. The constructs-only model (Model B2; AIC = 55.7) yielded comparable fit; PLF Awareness was the only psychological predictor of veterinarian-reported current client PLF use to reach statistical significance under conventional maximum-likelihood standard errors (*OR* = 3.07, 95% CI: 1.09–8.62, *p* = 0.034). However, under HC3 heteroscedasticity-consistent standard errors, this association attenuated to non-significance (Coef = 1.349, robust SE = 0.944, *z* = 1.43, *p*_robust = 0.153). The PLF Awareness association is therefore presented as an exploratory finding requiring confirmation in larger samples, consistent with the small-sample fragility of these estimates.

**Table 5:** Model comparison.

Model	*N*	AIC	McFadden *R*^2^	Nagelkerke *R*^2^
A1: demographics only	39	45.2	0.117	0.180
A2: constructs only	39	42.7	0.271	0.385
A3: full model	39	44.1	0.380	0.510
A4: full + client use	39	44.0	0.430	0.562
B1: demographics only	39	54.4	0.121	0.204
B2: constructs only	39	55.7	0.172	0.280
B3: full model	39	56.4	0.273	0.416

Lower Akaike Information Criterion (AIC) indicates better fit.

R^2^ values are pseudo-R^2^ for Logistic Regression (LR).

LR tests compare nested models.

Outcome A (veterinarian intention to recommend more PLF).

Outcome B (clients currently using PLF).

Four logistic regression models examined factors predicting veterinarians' intention to recommend PLF technologies to their clients (Table 6). Model A1 included only demographic predictors and explained modest variance (McFadden *R*^2^ = 0.117), with veterinarians who identified as general practitioner (GP) being the only significant predictor—GPs were about seven times more likely to intend to recommend PLF compared to non-GPs (*OR* = 6.94, *p* < 0.05). Model A2 included only five constructs and explained considerably more variance (McFadden *R*^2^ = 0.271), with PLF cost perception emerging as a marginal positive predictor (*OR* = 3.63, *p* < 0.10), suggesting that veterinarians who viewed PLF as cost-effective were more inclined to recommend it, though no predictor reached conventional significance. Model A3 combined demographic and the five constructs and explained the most variance of the first three models (McFadden *R*^2^ = 0.380), with general practitioner status again emerging as a marginally significant positive predictor (*OR* = 16.74, *p* < 0.10); none of the five constructs reached significance in this combined model. Model A4 added whether the veterinarian's clients currently use PLF and explained the greatest variance overall (McFadden *R*^2^ = 0.430), with general practitioner status remaining a marginal positive predictor (*OR* = 22.36, *p* < 0.10) and current client PLF use showing a strong but non-significant positive trend (*OR* = 8.45). Across these exploratory models, general practitioner status produced the largest and most consistent point estimates for recommendation intention; however, given the small sample, these are best interpreted as exploratory trends requiring confirmation in larger samples rather than as established or reliable predictors. None of the psychological constructs reached conventional statistical significance in the multivariable models for Outcome A. A forest plot of Model A3 odds ratios is provided in Supplementary Figure S8.

**Table 6:** Models A1-A4: factors predicting veterinarian intention to recommend more PLF.

Predictor	Model A1	Model A2	Model A3	Model A4
	OR	OR	OR	OR
Intercept	0.748	0.000^†^	0.000^*^	0.000^†^
PLF awareness		1.863	1.253	0.897
Expected PLF impact		1.626	4.096	8.744
PLF cost perception		3.628^†^	4.269	3.731
PLF subjective norms		0.932	1.020	1.187
PLF help wanted		2.429	1.615	0.927
Clients currently use PLF				8.451
Experience (years)	0.994		1.043	1.059
Male (ref: other)	1.390		1.111	0.311
General practitioner	6.944^*^		16.735^†^	22.363^†^
AIC	45.2	42.7	44.1	44.0
McFadden *R*^2^	0.117	0.271	0.380	0.430
Nagelkerke *R*^2^	0.180	0.385	0.510	0.562
*N*	39	39	39	39

OR, odds ratio;

^*^p < 0.05;^†^*p* < 0.10

#### 3.7.2 Predictors of veterinarian-reported client PLF use (outcome B)

Three logistic regression models examined factors predicting veterinarian-reported client PLF use (Table 7). Model B1 included only demographic predictors and explained modest variance (McFadden *R*^2^ = 0.121), finding that male veterinarian's clients were marginally more likely to use PLF compared to others (*OR* = 5.68, *p* < 0.10). Model B2 included only the five PLF constructs and explained slightly more variance (McFadden *R*^2^ = 0.172), with PLF Awareness as the only psychological predictor reaching conventional statistical significance under maximum-likelihood standard errors (*OR* = 3.07, *p* < 0.05); under HC3 robust standard errors this association attenuated to non-significance (*z* = 1.43, *p*_robust = 0.153). Model B3 combined both sets of predictors and explained the most variance overall (McFadden *R*^2^ = 0.273); PLF Awareness retained a positive point estimate (*OR* = 3.85, *p* < 0.05 under standard SE; *p*_robust = 0.153 under HC3) and being male showed a positive trend (*OR* = 8.03, *p* < 0.10 under standard SE; *p*_robust = 0.303 under HC3) that did not survive HC3 adjustment. Across all three models, no other predictors—including expected PLF impact, cost perception, subjective norms, wanting help with PLF, years of experience, or being a general practitioner—reached statistical significance. A forest plot of Model B3 odds ratios is provided in Supplementary Figure S9.

**Table 7:** Models B1 - B3: Factors predicting veterinarian-reported client PLF use.

Predictor	Model B1	Model B2	Model B3
	*OR*	*OR*	*OR*
(Intercept)	0.476	0.041	0.024
PLF awareness		3.066^*^	3.854^*^
Expected PLF impact		0.472	0.364
PLF cost perception		0.931	1.035
PLF subjective norms		0.498	0.580
PLF help wanted		3.373	3.391
Experience (years)	0.997		0.985
Male (ref: other)	5.680^†^		8.030^†^
General practitioner	1.911		0.443
AIC	54.4	55.7	56.4
McFadden *R*^2^	0.121	0.172	0.273
Nagelkerke *R*^2^	0.204	0.280	0.416
*N*	39	39	39

OR, odds ratio; ^*^p < 0.05;^†^*p* < 0.10.

### 3.8 Subgroup comparisons: construct scores by outcome group

Mann–Whitney *U*-tests comparing construct scores by outcome group are reported in Table 2. Veterinarians who planned to recommend PLF scored significantly higher on Expected PLF Impact (*W* = 476.5, *p* < 0.001), PLF Cost Perception (*W* = 486.0, *p* < 0.001), PLF Subjective Norms (*W* = 379.5, *p* = 0.014), and PLF Help Wanted (*W* = 386.0, *p* = 0.016) than those who did not plan to recommend. PLF Awareness did not significantly differentiate the two groups (*W* = 372.5, *p* = 0.100). Notably, no construct score differed significantly between veterinarians whose clients currently use vs. do not use PLF (all *p* > 0.16), indicating that psychological constructs are stronger discriminators of future recommendation intention than of observed current client adoption. Construct score distributions by outcome group are illustrated in Supplementary Figure S7.

### 3.9 Model diagnostics and robustness checks

VIF values for Model A3 were all below 5.00, indicating that multicollinearity was not a material concern for Outcome A (39). The Hosmer–Lemeshow goodness-of-fit test for Model A3 indicated adequate calibration (64). Cook's distance analysis identified influential observations (threshold: 4/*n* = 0.103) in Model A3 (Supplementary Figure S10) and Model B3 (Supplementary Figure S11); their removal in sensitivity analyses did not materially alter the direction of coefficient estimates. Subscale-based sensitivity regressions are reported in Supplementary Tables S9, S10.

The VIF values for Model B3 were also below 5.00, with no evidence of complete separation following the exclusion of location variables. HC3 robust standard errors were, as expected, larger than conventional maximum-likelihood standard errors in this small sample (62), and the directional pattern of associations remained consistent. Importantly, however, the robust SE results meaningfully attenuated the statistical significance of two findings: in Model A3, the marginal General Practitioner effect (*OR* = 16.74, *p* = 0.078 under standard SE) was reduced to non-significance under HC3 (Coef = 2.818, robust SE = 1.917, *z* = 1.47, *p*_robust = 0.142); and in Model B3, the previously significant PLF Awareness effect (*OR* = 3.85, *p* = 0.036 under standard SE) similarly attenuated under HC3 (Coef = 1.349, robust SE = 0.944, *z* = 1.43, *p*_robust = 0.153). The marginal Male trend in Model B1 (*p* = 0.078) likewise lost marginal status under HC3 (*p*_robust = 0.303). Full HC3 robust SE coefficient tables for Models A3 and B3 are provided in Supplementary Tables S3, S4. Firth penalized estimates (Supplementary Tables S5, S6) and multiple-imputation pooled estimates (Supplementary Tables S7, S8) yielded directionally consistent conclusions, with PLF Awareness in Model B3 attenuating under MI (pooled *p* = 0.095) as it did under HC3. These attenuations should not be interpreted as evidence that the underlying associations are absent; rather, they reflect the small-sample fragility of standard-error estimates and reinforce the framing of the multivariable models as exploratory.

## 4 Discussion

This study provides, to our knowledge, the first comprehensive psychometric and regression-based analysis of U.S. swine veterinarians' awareness of, attitudes toward, and intention to recommend PLF technologies, framed within the Theory of Planned Behavior. The findings reveal a nuanced picture: veterinarians in the U.S. swine sector are generally aware of PLF technologies, hold moderately positive evaluative attitudes, and show strong collective intention to recommend PLF to clients—yet their perceptions reflect important heterogeneities that carry practical implications for PLF extension programs and professional development efforts.

### 4.1 Construct validity and the five-construct model

All five constructs demonstrated good to excellent internal consistency (α = 0.796–0.896), justifying their use as composite predictors in regression analyses. The strong un-dimensionality of PLF Cost Perception (76.7% variance explained) and Expected PLF Impact (52.4%) is particularly noteworthy, as it suggests these evaluative dimensions are coherently operationalized in this population. The bifactorial structures of PLF Subjective Norms and PLF Help Wanted, each capturing two meaningful sub-dimensions, provide additional insight into the structure of veterinary attitudes toward PLF that simple single-factor models would not have revealed. Importantly, ω coefficients were commensurate with α, corroborating scale reliability and reducing concerns about violation of the tau-equivalence assumption underlying Cronbach's α (36, 37).

### 4.2 Awareness of PLF technologies and its implications

The high awareness scores for RFID-based identification (*M* = 4.69) and Electronic Sow Feeders (*M* = 4.51) are consistent with the long commercial history of these technologies in U.S. swine production (4, 6). By contrast, relatively low awareness of SowSIS pressure-plate lameness detection (*M* = 2.62) and individual water intake monitoring (*M* = 2.80) points to a knowledge gap for technologies that are newer to the commercial market or were developed predominantly in European production contexts (28, 29). This pattern aligns with Rogers' (22) diffusion of innovations framework, in which awareness-knowledge precedes adoption but diffuses unevenly depending on commercial availability, geographical reach, and coverage in professional training programs.

From a practical standpoint, the substantial variance in awareness across technologies suggests that differentiated educational strategies are warranted. Continuing professional education programs, such as those delivered through the American Association of Swine Veterinarians (AASV), are well-positioned to close awareness gaps for less-familiar technologies. This approach is consistent with the broader precision agriculture adoption literature, which identifies targeted knowledge delivery as the most cost-effective mechanism for shifting awareness into active evaluation of new tools (69–71), particularly for precision lameness and water intake monitoring tools with strong animal welfare relevance (4).

### 4.3 Expected PLF impact, cost perception, and their interrelation

Expected PLF Impact (*M* = 3.78) and PLF Cost Perception (*M* = 2.95) together reflect a profile of positive outcome expectations tempered by genuine financial ambivalence. The strong positive Spearman correlation between these constructs (ρ = 0.711, *p* < 0.001) indicates that veterinarians who perceive PLF as highly impactful tend to judge the financial investment as justified—a value-for-money reasoning pattern analogous to that documented in precision dairy farming adoption studies (72). This correlation also suggests that the two constructs do not function as independent drivers of recommendation intention; rather, veterinarians appear to weigh expected benefits against perceived costs as a bundled value assessment.

In the regression analyses, Expected PLF Impact showed the largest positive point estimate in Model A3 (*OR* = 4.096), consistent with the Technology Acceptance Model's finding that perceived usefulness is the dominant predictor of adoption intention (73). This finding reinforces the practical priority of enhancing veterinarians' outcome expectations through evidence-based case studies, on-farm demonstration events, and targeted communication of validated PLF efficacy data (3, 4).

### 4.4 Subjective norms, social influence, and PLF help wanted

The PLF Subjective Norms scale revealed modest perceived social pressure to recommend PLF (*M* = 3.11). The two EFA sub-dimensions—informal/commercial channels (social media, client, and industry representatives) and formal professional channels (colleagues and professional associations)—are substantively informative. The relatively lower loading from formal professional organizations may reflect that, at the time of data collection (2022–2023), the AASV and comparable bodies had not yet issued systematic guidance on PLF recommendation. This structural gap represents a modifiable target: professional associations can meaningfully increase PLF recommendation norms by developing evidence-based position statements, continuing education curricula, and accreditation for PLF competencies (10).

The significant positive association between PLF Subjective Norms and PLF Help Wanted (ρ = 0.334, *p* = 0.010) suggests that veterinarians who perceive greater social endorsement of PLF also recognize broader client need for PLF-based solutions, consistent with social learning processes whereby normative information shapes the perceived relevance of an innovation (22, 74). PLF Help Wanted emerged as the highest-rated construct overall (*M* = 3.91), indicating strong perceived client demand for PLF assistance across production problem areas including lameness, tail biting (75), aggression (76), piglet mortality, and respiratory disease. Notably, these are precisely the welfare domains that the Common Swine Industry Audit (CSIA) identifies as critical failure criteria and animal-based measures requiring daily stockperson assessment—domains for which Benjamin and Yik (7) describe validated and emerging PLF monitoring tools as directly applicable.

### 4.5 Geographical variation in veterinarian-reported client PLF use

A chi-square test indicated that veterinarian-reported client PLF use varied by practice location (χ^2^ = 13.79, *p* = 0.003). These descriptive geographical differences most plausibly reflect structural differences in swine production systems: Iowa is the leading U.S. pork-producing state with a high concentration of large, vertically integrated operations in which PLF adoption is more economically viable and has been actively promoted by integrators (1). North Carolina's swine industry, while also substantial, has faced significant regulatory scrutiny and community-level opposition related to environmental and odor concerns that may have redirected producer and veterinary attention away from technology investment (77). These industry-level explanations are offered as hypothesis-generating context for the descriptive geographic pattern rather than as conclusions directly supported by the present data. Importantly, location indicators were excluded from regression models because the small sample (*N* = 61) produced near-perfect separation, generating unreliable odds ratios. Near-perfect separation occurs when one or more covariate values almost perfectly predict the binary outcome, leaving the maximum-likelihood estimator without a finite optimum and producing implausibly large odds ratios and very wide confidence intervals (51, 53). This underscores the need for larger, regionally stratified samples to formally quantify the independent contribution of geographic context to client PLF adoption. In the demographics-only regression model (B1), gender showed only a marginal non-significant trend toward higher recommendation odds for male veterinarians (*p* = 0.078); larger samples are required before substantive interpretation is appropriate.

### 4.6 Recommendation intention vs. veterinarian-reported client PLF use

The hierarchical regression analyses reveal an exploratory asymmetry: psychological constructs more clearly differentiate veterinarians by recommendation intention than by veterinarian-reported current client PLF use. It is important to distinguish bivariate group differences from independent effects in adjusted multivariable models. In bivariate Mann–Whitney comparisons, four of five constructs differentiated veterinarians intending vs. not intending to recommend PLF (all uncorrected *p* ≤ 0.016); however, in the adjusted multivariable Model A3, none of the psychological constructs reached conventional statistical significance, and effect estimates should be regarded as imprecise given the small sample. Conversely, none of the bivariate construct comparisons reached significance for veterinarian-reported current client PLF use (all *p* > 0.16). This pattern is consistent with the possibility that recommendation intention is shaped by the veterinarian's own evaluative beliefs and social perceptions, while veterinarian-reported client PLF use may also reflect contextual and structural factors—such as regional production system characteristics—that operate beyond individual psychology. The directionality of the recommendation–adoption relationship cannot be determined from these cross-sectional data: it is plausible that veterinarian recommendation increases client adoption, that working with PLF-adopting clients shapes veterinarian intention, or that both are jointly driven by upstream factors (e.g., integrator policies, regional infrastructure). Where psychological constructs are weakly linked to current client PLF use, the role of veterinarians as decision-shapers—rather than passive observers—in PLF adoption deserves direct longitudinal investigation. We further note that in Model B2/B3 the conventionally significant PLF Awareness effect attenuated to non-significance under HC3 heteroscedasticity-consistent robust standard errors (*p*_robust = 0.153), and the marginal General Practitioner effect in Model A3 (*p* = 0.078) similarly lost significance under HC3 (*p*_robust = 0.142). These attenuations indicate that the standard-error estimates are themselves fragile in this small sample and reinforce the exploratory framing of all multivariable findings.

The finding that adding veterinarian-reported current client PLF use to the full regression model provided no incremental predictive value (LR *p* = 0.945) further reinforces this interpretation: attitudinal interventions targeting veterinarian beliefs and norms may be effective even among veterinarians who do not yet serve clients using PLF, potentially positioning these practitioners as early advisors rather than laggards in PLF diffusion.

The absence of statistical significance for individual predictors in model A3 should be understood as a power limitation rather than evidence of negligible effects. With *n* = 39, the study was substantially underpowered for detecting moderate effect sizes in logistic regression (78). Future studies should prioritize samples of large size to reliably estimate individual predictor effects.

### 4.7 Strengths, limitations, and future directions

This study has several notable strengths. The application of a validated theoretical framework (TPB), the use of rigorous construct validation procedures (parallel analysis, EFA, α and ω reliability screening), and comprehensive regression diagnostics—including robust standard errors, Cook's distance analysis, and mean-imputation sensitivity checks—represent methodological rigor. Importantly, this study captures both intention (a psychological endpoint) and observed client behavior (a real-world adoption indicator), providing a broad picture of the veterinarian's role in PLF diffusion than either measure alone.

Several limitations must be acknowledged. First, the sample size (*N* = 61 total; *n* = 39 complete cases for regression) is small, limiting statistical power and the precision of individual predictor estimates. Second, the cross-sectional design precludes causal inference; longitudinal designs are needed to confirm that changes in attitudes and norms temporally precede changes in recommendation behavior, as the TPB requires. Third, convenience sampling through professional meeting networks may have over-represented veterinarians with pre-existing interest in PLF, introducing self-selection bias of unknown magnitude; meeting attendance counts were not systematically recorded, and a formal response rate could not therefore be computed, which limits the assessment of non-response bias and supports the framing of the sample as a convenience sample of U.S. swine veterinarians rather than a probability sample (79). Fourth, the in-meeting questionnaire context may have introduced demand characteristics or social desirability effects not present in individually administered surveys; the standardized PLF definition and presentation of commercially available examples, while improving consistency in respondents' understanding of PLF, may also have primed more positive evaluations and inflated estimated recommendation intention (80, 81). Self-reported intention is also susceptible to professional-norm-aligned response patterns in meeting settings, and the 74.5% intention rate should therefore be regarded as a context-bound estimate. Fifth, this study did not measure perceived behavioral control (PBC), a standard TPB component; future work should incorporate PBC items to complete the theoretical model. Sixth, Outcome B reflects veterinarian-reported client PLF use rather than directly observed farm-level adoption; this measure may be affected by the veterinarian's knowledge of client operations, client mix, frequency of contact, and recall, and should be interpreted accordingly. Seventh, the questionnaire did not include an item asking whether the veterinarian had previously recommended PLF to clients, which would have provided useful behavioral context alongside the prospective intention measure; we recommend including such an item in future surveys. Eighth, although the EFA suggested two-factor solutions for PLF Subjective Norms and PLF Help Wanted, the small sample limited the stability of these factor structures, and we use composite scores in regression with subscale-based sensitivity analyses; replication in larger samples is required before strong claims about subscale structure can be made (82). Ninth, the multivariable models include several predictors with limited events per variable, and we therefore complement the standard maximum-likelihood models with Firth penalized regression and report multiple-imputation and HC3 heteroscedasticity-consistent standard-error sensitivity analyses; under HC3 robust SE, the marginal General Practitioner effect in Model A3 and the conventionally significant PLF Awareness effect in Model B3 both attenuated to non-significance (*p*_robust = 0.142 and 0.153, respectively), reinforcing that all multivariable findings should be regarded as exploratory pending replication. We emphasize that even after Firth penalization, HC3 robust standard errors, and multiple-imputation pooling, statistical power remained limited: the sensitivity analyses confirm directional consistency rather than provide a substitute for adequate sample size, and all multivariable findings should accordingly be regarded as hypothesis-generating pending replication in larger, nationally representative samples. Tenth, multiple bivariate tests were performed; we report uncorrected *p*-values alongside Benjamini–Hochberg-adjusted *q*-values for transparency. Finally, the study's cross-sectional design and reliance on a single professional network limit generalizability to the broader U.S. swine veterinarian population.

A focused future research agenda—rather than an exhaustive one—would prioritize two practical questions raised by these findings. First, larger, nationally representative samples (e.g., recruited through the AASV membership database or in collaboration with AVMA-affiliated lists) would allow Firth-stabilized or Bayesian replications of the present exploratory models and direct measurement of perceived behavioral control, including self-efficacy in evaluating PLF technologies and communicating cost–benefit tradeoffs to producers. Second, and most importantly, future work should document whether specific PLF technologies actually deliver the welfare and productivity benefits attributed to them, and identify which advice channels and information sources are most influential in motivating producer adoption of those technologies that are demonstrably beneficial (9, 18). Linking veterinarian recommendation, producer adoption, and on-farm welfare outcomes would provide the policy-relevant evidence base on which sustained investment in veterinarian engagement with PLF should ultimately rest.

## 5 Conclusion

U.S. swine veterinarians show strong collective intent to recommend PLF technologies, with 74.5% expressing positive recommendation intention. Evaluative beliefs about PLF's expected impact and cost-effectiveness, perceived professional and social norms, and recognition of client need for PLF-based assistance are candidate modifiable correlates of that intention in this exploratory, partially TPB-grounded analysis. Cost perception sits near the scale midpoint and emerges as a recurring brake on recommendation intention; outreach, extension, and continuing-education efforts that explicitly address installation, operation, and maintenance costs—for example through return-on-investment case studies, validated benefit–cost evidence, and sliding-scale technology trials—are likely to yield the largest practical gains. Veterinarian-reported current client PLF use was not significantly predicted by any construct in bivariate subgroup comparisons. PLF Awareness was the only construct reaching conventional statistical significance under standard maximum-likelihood standard errors in the regression models for Outcome B, but this association attenuated to non-significance under HC3 robust standard errors and should therefore be regarded as exploratory. Observed client PLF adoption was not significantly predicted by any construct in the subgroup analyses, and practice location indicators could not be included in regression models due to near-perfect separation; descriptive evidence suggests that structural and regional factors warrant investigation in larger samples. These findings provide an actionable, theoretically grounded evidence base for professional associations, extension agencies, and technology developers seeking to engage swine veterinarians as change agents for responsible and welfare-oriented PLF adoption. The TPB constructs identified here as significant discriminators of recommendation intention—Expected PLF Impact, PLF Cost Perception, PLF Subjective Norms, and PLF Help Wanted—offer concrete, measurable targets for intervention design. Future research should prioritize larger, nationally representative samples, longitudinal designs, and integration of perceived behavioral control to fully test the TPB causal chain in this important professional context.

## Data Availability

The raw data supporting the conclusions of this article will be made available by the authors, without undue reservation.
