# Supplementary material for: Swine veterinarians' awareness, attitudes, and intention to recommend precision livestock farming technologies to clients
Source: Front Vet Sci. 2026 Jun 15;13:1841102. doi: 10.3389/fvets.2026.1841102 (PMC13310784; doi:10.3389/fvets.2026.1841102)
Supplement: Supplementary file 1 [file Data_Sheet_1.docx]

**Supplementary Material**

*Swine Veterinarians’ Awareness, Attitudes, and Intention to Recommend Precision Livestock Farming Technologies to Clients*

# Inter-Construct Correlations

Table S1 presents Spearman rank correlations among the five construct scores. The strongest association was between Expected PLF Impact and PLF Cost Perception (ρ = 0.711), indicating that veterinarians who anticipated greater positive PLF outcomes also judged the financial investment as more justifiable.

**Table S1. Spearman Rank Correlation Matrix — Construct Scores**

| Construct | PLF Awareness | Expected PLF Impact | PLF Cost Perception | PLF Subjective Norms | PLF Help Wanted |
| --- | --- | --- | --- | --- | --- |
| PLF Awareness | **1.000** | 0.269 | 0.194 | 0.300 | 0.193 |
| Expected PLF Impact | 0.269 | **1.000** | 0.711 | 0.288 | 0.185 |
| PLF Cost Perception | 0.194 | 0.711 | **1.000** | 0.331 | 0.147 |
| PLF Subjective Norms | 0.300 | 0.288 | 0.331 | **1.000** | 0.334 |
| PLF Help Wanted | 0.193 | 0.185 | 0.147 | 0.334 | **1.000** |

*Note. All values are Spearman’s ρ. Bold diagonal values (ρ = 1.000) indicate self-correlations.*

# Logistic Regression — Outcome A: Veterinarian Intention to Recommend More PLF

Four hierarchical binary logistic regression models were estimated. Tables S2–S5 report odds ratios (OR) with 95% confidence intervals and p-values for each model. Practice location was excluded from all models due to near-perfect separation in the small sample (N = 61), which produced unreliable odds ratios. An OR > 1 indicates a higher probability that the veterinarian will recommend more PLF.

## Model A1: Sociodemographic Predictors Only

**Table S2. Model A1 — Sociodemographic Predictors Only (Outcome A)**

| Predictor | OR | 95% CI Lower | 95% CI Upper | p-value | Sig. |
| --- | --- | --- | --- | --- | --- |
| (Intercept) | 0.748 | 0.112 | 4.996 | 0.7646 |  |
| Experience (yrs) | 0.994 | 0.898 | 1.100 | 0.9017 |  |
| Male (ref: other) | 1.390 | 0.154 | 12.586 | 0.7695 |  |
| General Practitioner | 6.944 | 1.162 | 41.496 | 0.0336 | * |

*Note. *** p < 0.001 ** p < 0.01 * p < 0.05 † p < 0.10; n = 39 | AIC = 45.2 | McFadden R² = 0.117 | Nagelkerke R² = 0.180;* OR = odds ratio; 95% CI = 95% confidence interval. Reference category for Male: female or prefer not to disclose. General Practitioner reference: other specialty.

## Model A2: TPB Construct Predictors Only

**Table S3. Model A2 — TPB Construct Predictors Only (Outcome A)**

| Predictor | OR | 95% CI Lower | 95% CI Upper | p-value | Sig. |
| --- | --- | --- | --- | --- | --- |
| (Intercept) | 0.000 | 0.000 | 1.015 | 0.0504 | † |
| PLF Awareness | 1.863 | 0.621 | 5.589 | 0.2669 |  |
| Expected PLF Impact | 1.626 | 0.170 | 15.598 | 0.6734 |  |
| PLF Cost Perception | 3.628 | 0.783 | 16.805 | 0.0994 | † |
| PLF Subjective Norms | 0.932 | 0.291 | 2.988 | 0.9054 |  |
| PLF Help Wanted | 2.429 | 0.490 | 12.051 | 0.2775 |  |

*Note. *** p < 0.001 ** p < 0.01 * p < 0.05 † p < 0.10; n = 39 | AIC = 42.7 | McFadden R² = 0.271 | Nagelkerke R² = 0.385;* OR = odds ratio; 95% CI = 95% confidence interval.

## Model A3: Full Model (Constructs + Socio-demographics)

**Table S4. Model A3 — Full Model (Outcome A)**

| Predictor | OR | 95% CI Lower | 95% CI Upper | p-value | Sig. |
| --- | --- | --- | --- | --- | --- |
| (Intercept) | 0.000 | 0.000 | 0.617 | 0.0420 | * |
| PLF Awareness | 1.253 | 0.354 | 4.433 | 0.7265 |  |
| Expected PLF Impact | 4.096 | 0.282 | 59.464 | 0.3016 |  |
| PLF Cost Perception | 4.269 | 0.734 | 24.822 | 0.1061 |  |
| PLF Subjective Norms | 1.020 | 0.190 | 5.481 | 0.9813 |  |
| PLF Help Wanted | 1.615 | 0.309 | 8.452 | 0.5701 |  |
| Experience (yrs) | 1.043 | 0.880 | 1.235 | 0.6278 |  |
| Male (ref: other) | 1.111 | 0.037 | 33.127 | 0.9516 |  |
| General Practitioner | 16.735 | 0.733 | 382.184 | 0.0775 | † |

*Note. *** p < 0.001 ** p < 0.01 * p < 0.05 † p < 0.10; n = 39 | AIC = 44.1 | McFadden R² = 0.380 | Nagelkerke R² = 0.510;* OR = odds ratio; 95% CI = 95% confidence interval. A forest plot of Model A3 odds ratios is provided in Supplementary Figure S8.

## Model A4: Full Model + Client PLF Use

**Table S5. Model A4 — Full Model + Client PLF Use (Outcome A)**

| Predictor | OR | 95% CI Lower | 95% CI Upper | p-value | Sig. |
| --- | --- | --- | --- | --- | --- |
| (Intercept) | 0.000 | 0.000 | 1.158 | 0.0524 | † |
| PLF Awareness | 0.897 | 0.219 | 3.666 | 0.8794 |  |
| Expected PLF Impact | 8.744 | 0.379 | 201.957 | 0.1759 |  |
| PLF Cost Perception | 3.731 | 0.649 | 21.445 | 0.1400 |  |
| PLF Subjective Norms | 1.187 | 0.184 | 7.651 | 0.8572 |  |
| PLF Help Wanted | 0.927 | 0.130 | 6.615 | 0.9394 |  |
| Clients Currently Use PLF | 8.451 | 0.374 | 191.030 | 0.1798 |  |
| Experience (yrs) | 1.059 | 0.891 | 1.259 | 0.5127 |  |
| Male (ref: other) | 0.311 | 0.007 | 13.873 | 0.5470 |  |
| General Practitioner | 22.363 | 0.614 | 814.275 | 0.0902 | † |

*Note. *** p < 0.001 ** p < 0.01 * p < 0.05 † p < 0.10; n = 39 | AIC = 44.0 | McFadden R² = 0.430 | Nagelkerke R² = 0.562;* OR = odds ratio; 95% CI = 95% confidence interval. Adding current client PLF use provided no meaningful incremental improvement over Model A3 (ΔAIC = 0.1).

Logistic Regression — Outcome B: Clients Currently Using PLF

Three hierarchical binary logistic regression models were estimated for the secondary outcome (whether the veterinarian reports that at least some of their clients currently use PLF). Tables S6–S8 report OR with 95% confidence intervals and p-values.

## Model B1: Sociodemographic Predictors Only

**Table S6. Model B1 — Sociodemographic Predictors Only (Outcome B)**

| Predictor | OR | 95% CI Lower | 95% CI Upper | p-value | Sig. |
| --- | --- | --- | --- | --- | --- |
| (Intercept) | 0.476 | 0.072 | 3.141 | 0.4406 |  |
| Experience (yrs) | 0.997 | 0.914 | 1.087 | 0.9383 |  |
| Male (ref: other) | 5.680 | 0.824 | 39.166 | 0.0779 | † |
| General Practitioner | 1.911 | 0.321 | 11.373 | 0.4766 |  |

*Note. *** p < 0.001 ** p < 0.01 * p < 0.05 † p < 0.10; n = 39 | AIC = 54.4 | McFadden R² = 0.121 | Nagelkerke R² = 0.204;* OR = odds ratio; 95% CI = 95% confidence interval.

## Model B2: TPB Construct Predictors Only

**Table S7. Model B2 — TPB Construct Predictors Only (Outcome B)**

| Predictor | OR | 95% CI Lower | 95% CI Upper | p-value | Sig. |
| --- | --- | --- | --- | --- | --- |
| (Intercept) | 0.041 | 0.000 | 32.663 | 0.3482 |  |
| PLF Awareness | 3.066 | 1.090 | 8.625 | 0.0337 | * |
| Expected PLF Impact | 0.472 | 0.063 | 3.548 | 0.4658 |  |
| PLF Cost Perception | 0.931 | 0.300 | 2.890 | 0.9019 |  |
| PLF Subjective Norms | 0.498 | 0.201 | 1.236 | 0.1326 |  |
| PLF Help Wanted | 3.373 | 0.785 | 14.498 | 0.1022 |  |

*Note. *** p < 0.001 ** p < 0.01 * p < 0.05 † p < 0.10; n = 39 | AIC = 55.7 | McFadden R² = 0.172 | Nagelkerke R² = 0.280;* OR = odds ratio; 95% CI = 95% confidence interval.

## Model B3: Full Model (Constructs + Socio-demographics)

**Table S8. Model B3 — Full Model (Outcome B)**

| Predictor | OR | 95% CI Lower | 95% CI Upper | p-value | Sig. |
| --- | --- | --- | --- | --- | --- |
| (Intercept) | 0.024 | 0.000 | 44.876 | 0.3327 |  |
| PLF Awareness | 3.854 | 1.094 | 13.578 | 0.0357 | * |
| Expected PLF Impact | 0.364 | 0.039 | 3.391 | 0.3745 |  |
| PLF Cost Perception | 1.035 | 0.322 | 3.325 | 0.9544 |  |
| PLF Subjective Norms | 0.580 | 0.222 | 1.517 | 0.2668 |  |
| PLF Help Wanted | 3.391 | 0.655 | 17.559 | 0.1455 |  |
| Experience (yrs) | 0.985 | 0.885 | 1.096 | 0.7768 |  |
| Male (ref: other) | 8.030 | 0.754 | 85.514 | 0.0843 | † |
| General Practitioner | 0.443 | 0.046 | 4.307 | 0.4831 |  |

*Note. *** p < 0.001 ** p < 0.01 * p < 0.05 † p < 0.10; n = 39 | AIC = 56.4 | McFadden R² = 0.273 | Nagelkerke R² = 0.416;* OR = odds ratio; 95% CI = 95% confidence interval. A forest plot of Model B3 odds ratios is provided in Supplementary Figure S9.

Model Diagnostics — HC3 Heteroscedasticity-Consistent Robust Standard Errors

HC3 robust standard errors (Long and Ervin, 2000; Zeileis, 2004) were computed as a robustness check for the full models. Robust SE estimates are, as expected, larger than conventional maximum-likelihood standard errors in this small sample, but the directional pattern of associations remained consistent.

## HC3 Robust Standard Errors — Model A3

**Table S9. HC3 Robust Standard Errors — Model A3 (Outcome A)**

| Predictor | Coef. | Robust SE | z | p (robust) |
| --- | --- | --- | --- | --- |
| (Intercept) | −13.304 | 9.050 | −1.47 | 0.1415 |
| PLF Awareness | 0.226 | 1.335 | 0.17 | 0.8659 |
| Expected PLF Impact | 1.410 | 2.500 | 0.56 | 0.5726 |
| PLF Cost Perception | 1.451 | 1.713 | 0.85 | 0.3967 |
| PLF Subjective Norms | 0.020 | 1.880 | 0.01 | 0.9915 |
| PLF Help Wanted | 0.480 | 1.631 | 0.29 | 0.7688 |
| Experience (yrs) | 0.042 | 0.096 | 0.43 | 0.6636 |
| Male (ref: other) | 0.105 | 2.834 | 0.04 | 0.9704 |
| General Practitioner | 2.818 | 1.917 | 1.47 | 0.1417 |

*Note.* Coef. = log-odds coefficient. Robust SE computed using the HC3 estimator (sandwich package in R). Cook’s distance diagnostics for Model A3 are provided in Supplementary Figure S10.

## HC3 Robust Standard Errors — Model B3

**Table S10. HC3 Robust Standard Errors — Model B3 (Outcome B)**

| Predictor | Coef. | Robust SE | z | p (robust) |
| --- | --- | --- | --- | --- |
| (Intercept) | −3.717 | 5.285 | −0.70 | 0.4819 |
| PLF Awareness | 1.349 | 0.944 | 1.43 | 0.1529 |
| Expected PLF Impact | −1.011 | 1.420 | −0.71 | 0.4764 |
| PLF Cost Perception | 0.034 | 1.061 | 0.03 | 0.9744 |
| PLF Subjective Norms | −0.545 | 0.708 | −0.77 | 0.4417 |
| PLF Help Wanted | 1.221 | 1.082 | 1.13 | 0.2591 |
| Experience (yrs) | −0.015 | 0.089 | −0.17 | 0.8621 |
| Male (ref: other) | 2.083 | 2.023 | 1.03 | 0.3031 |
| General Practitioner | −0.814 | 1.492 | −0.55 | 0.5857 |

*Note.* Coef. = log-odds coefficient. Robust SE computed using the HC3 estimator (sandwich package in R). Cook’s distance diagnostics for Model B3 are provided in Supplementary Figure S11.

# **Figures**


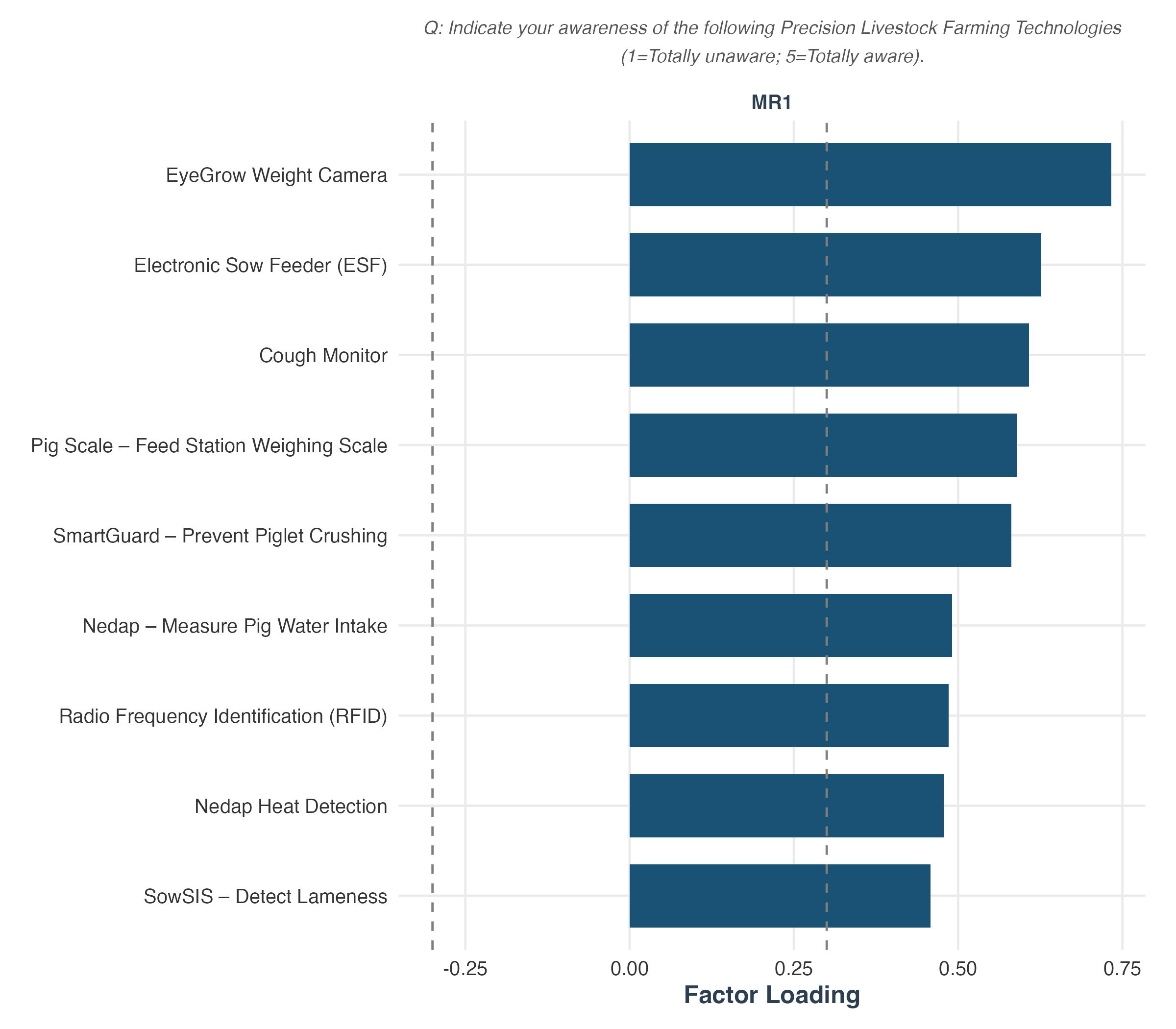


**Figure S1. EFA Factor Loadings — PLF Awareness (MR1)**

*Note.* Exploratory factor analysis ( n = 61). Item-level factor loadings on the single retained factor (MR1) are displayed in descending order. The solid grey reference line marks zero; the dashed grey line marks the 0.32 minimum loading threshold. All nine PLF technology items loaded positively on MR1 (range: 0.46–0.75), supporting a unidimensional awareness construct (α = 0.796, cumulative variance = 32.2%). Scale: 1 = Totally Unaware to 5 = Totally Aware.


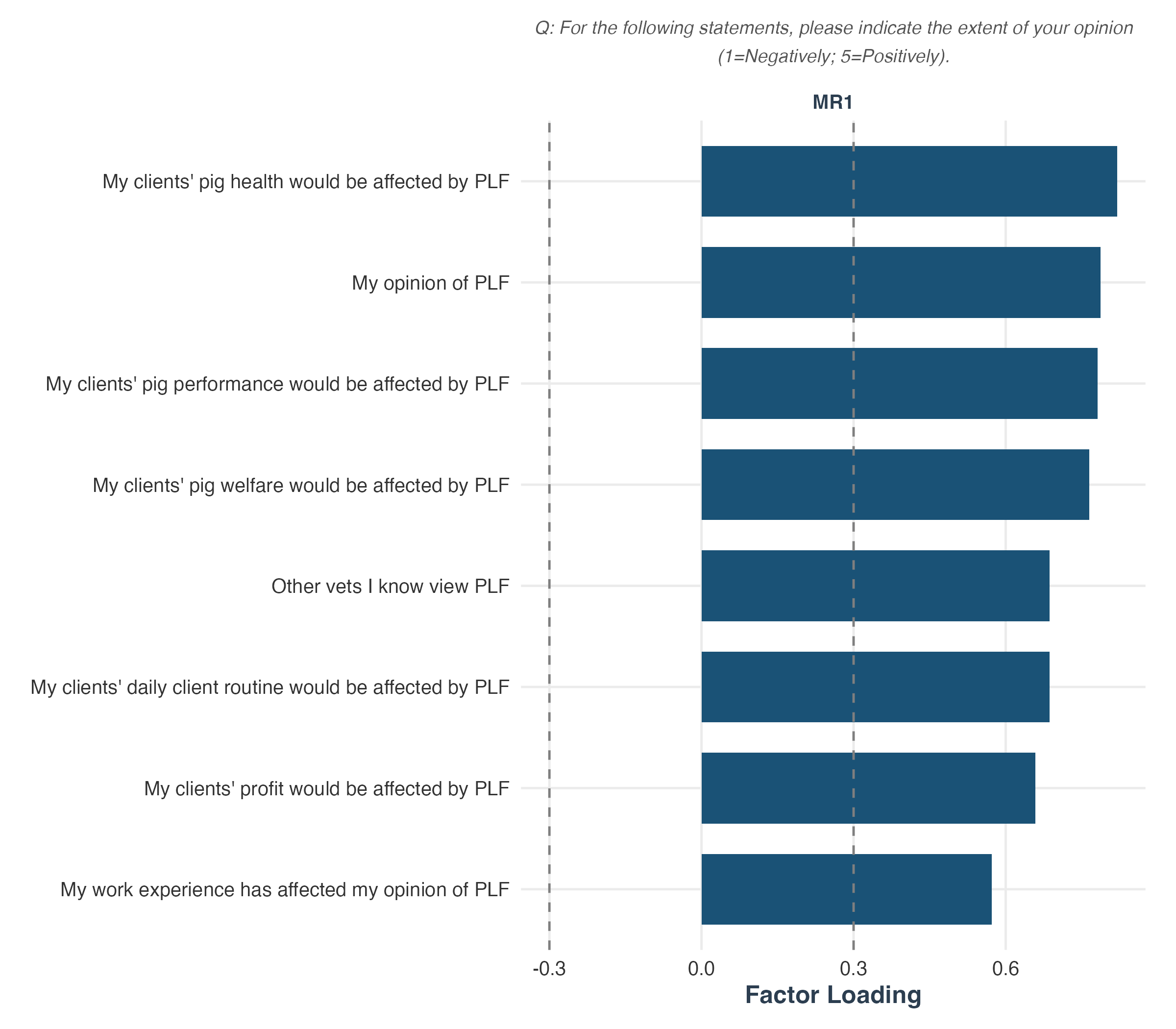


**Figure S2. EFA Factor Loadings — Expected PLF Impact (MR1)**

*Note.* Exploratory factor analysis (n = 61). Item-level factor loadings on the single retained factor (MR1) are displayed in descending order. All eight items loaded positively on MR1 (range: 0.56–0.84), supporting a unidimensional construct reflecting veterinarians’ evaluative beliefs about PLF’s effect on pig health, welfare, performance, profitability, and the veterinarian’s own work experience (α = 0.893, cumulative variance = 52.4%). Scale: 1 = Negatively to 5 = Positively.


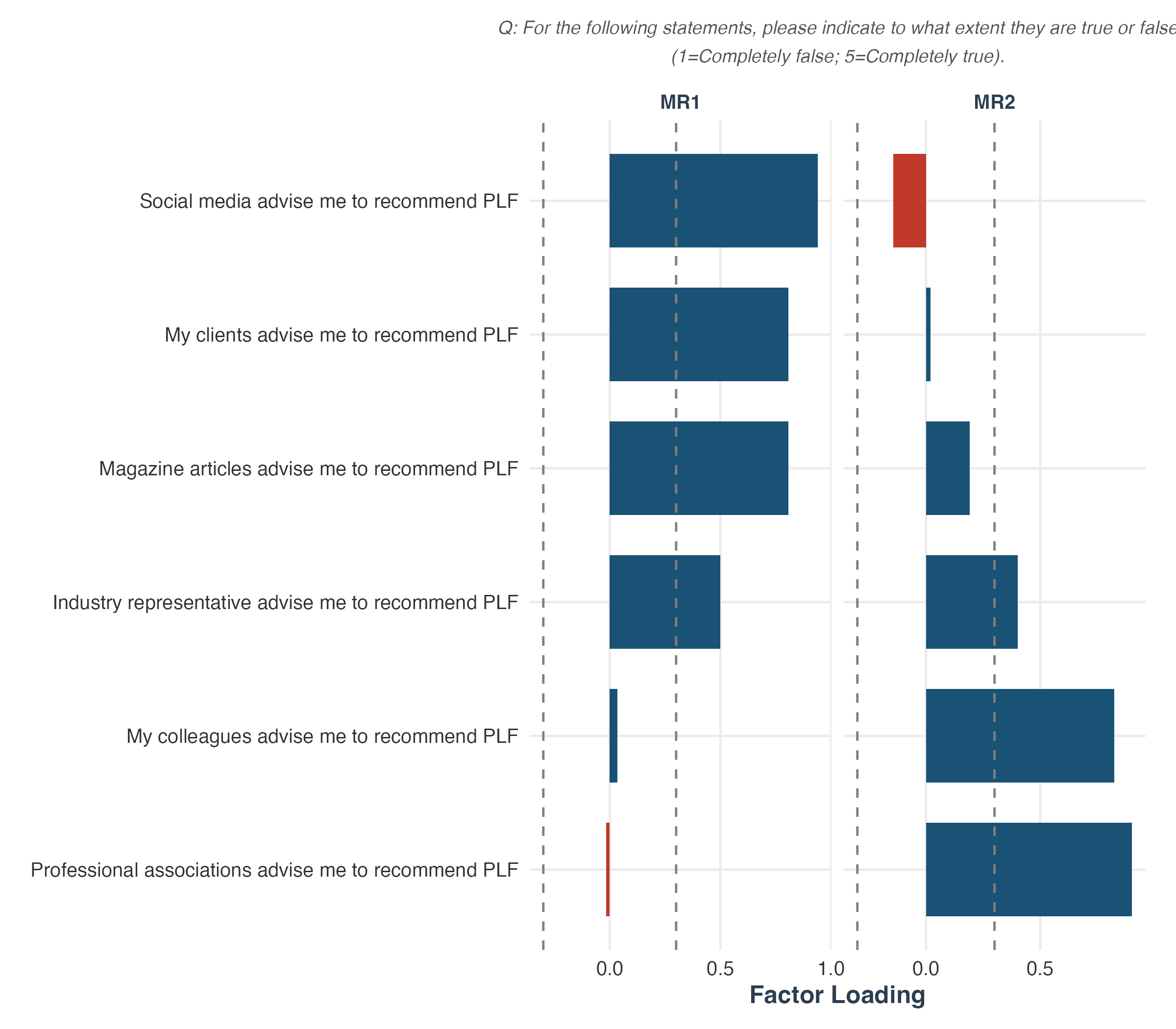


**Figure S3. EFA Factor Loadings — PLF Subjective Norms (MR1 and MR2)**

*Note.* Exploratory factor analysis (n = 59). Two factors were retained (cumulative variance = 73.5%). MR1 (informal/commercial channels) captures perceived pressure from social media, clients, magazine articles, and industry representatives to recommend PLF. MR2 (formal/professional channels) captures perceived pressure from colleagues and professional associations. Red bars indicate cross-loadings exceeding the 0.32 threshold on the secondary factor. α = 0.884 for the composite scale. Scale: 1 = Completely False to 5 = Completely True.


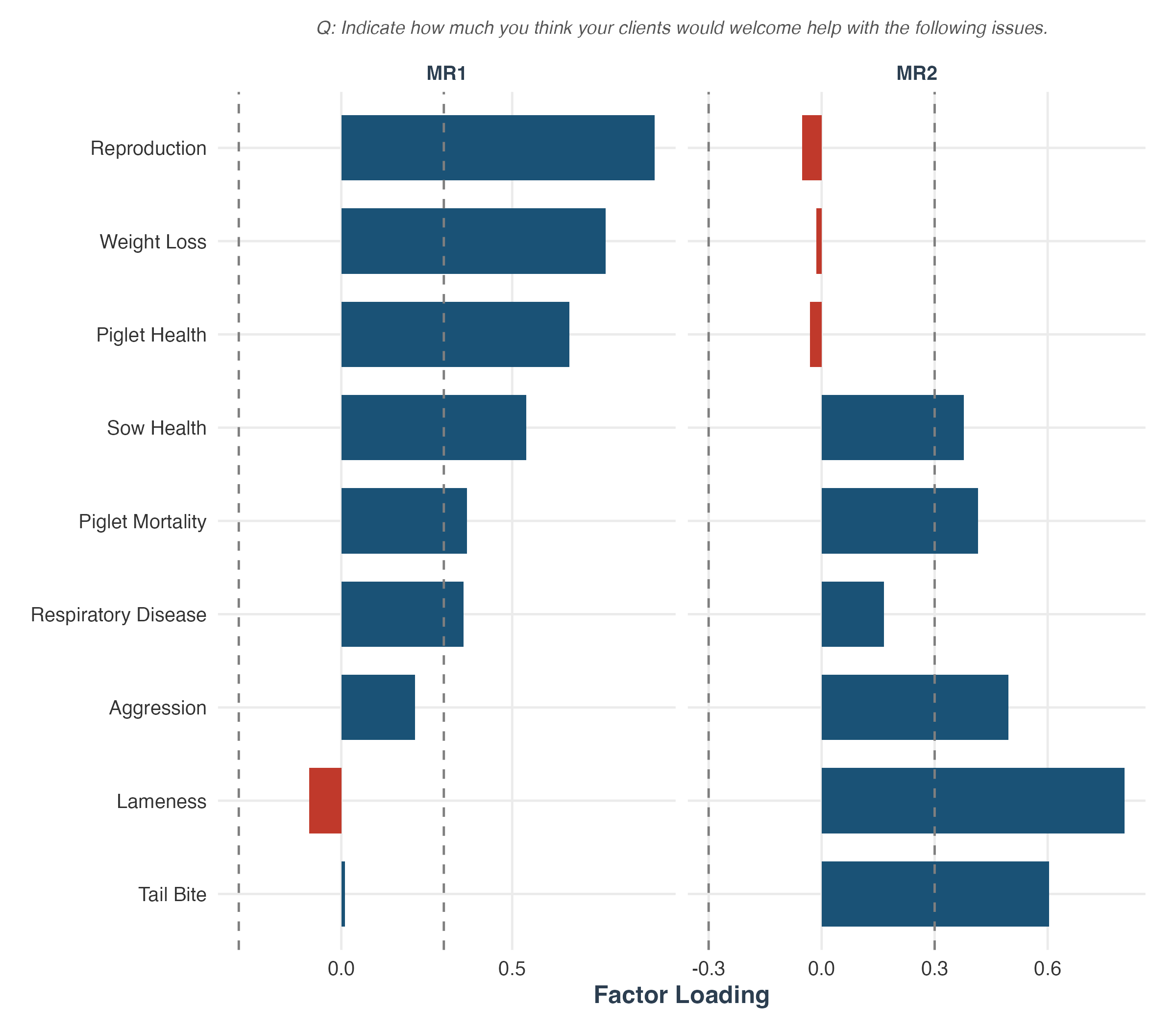


**Figure S4. EFA Factor Loadings — PLF Help Wanted (MR1 and MR2)**

*Note.* Exploratory factor analysis (n = 61). Two factors were retained (cumulative variance = 49.0%). MR1 (sow-level and reproductive problems) captures client-perceived need for PLF assistance with reproduction, weight loss, piglet health, sow health, piglet mortality, respiratory disease, and aggression. MR2 (group/pen-level problems) captures need for PLF assistance with lameness and tail biting. Red bars indicate cross-loadings exceeding the 0.32 threshold on the secondary factor. α = 0.837 for the composite scale. Scale: 1 = No Help Wanted to 5 = A Lot of Help Wanted.


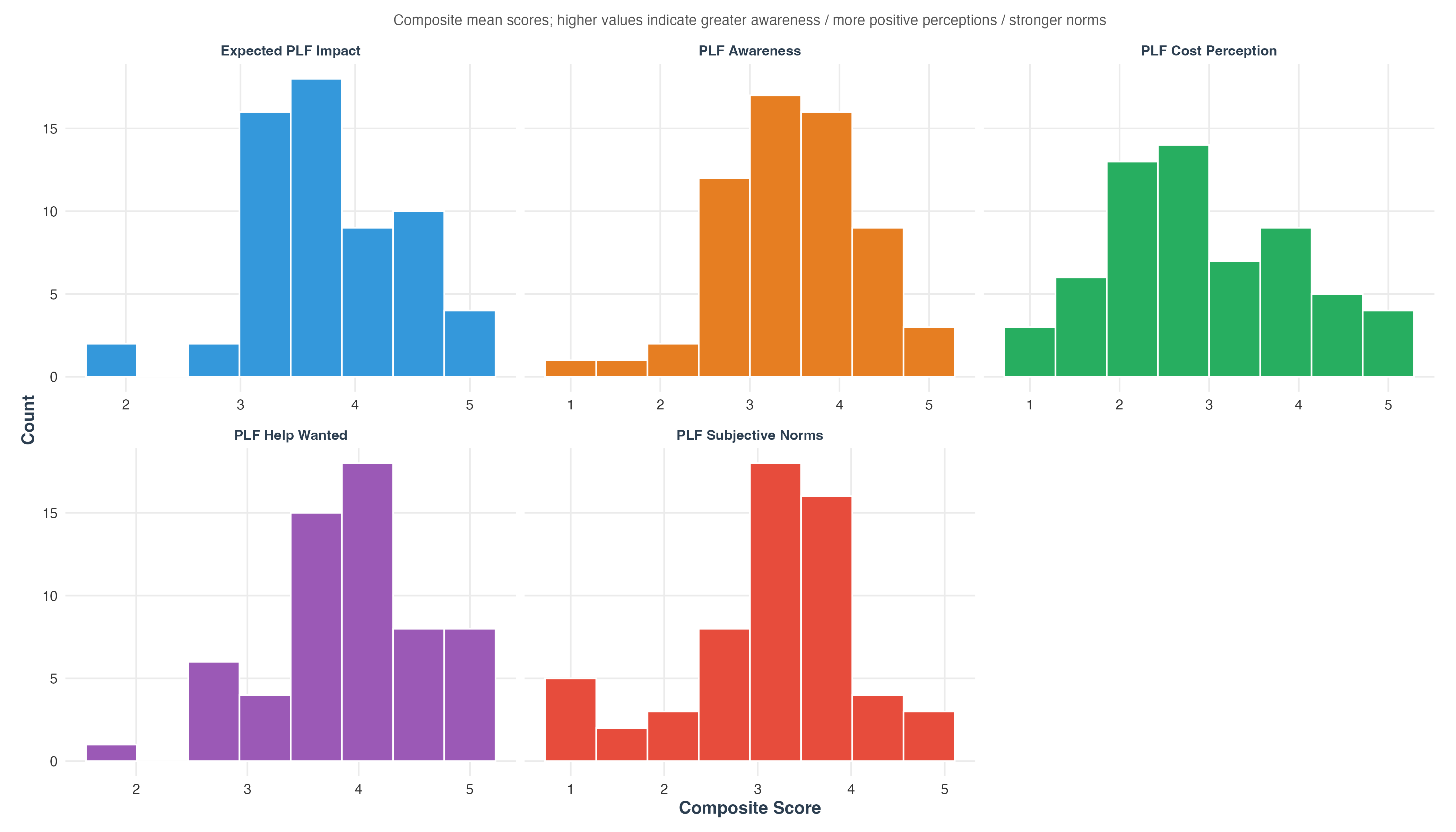


**Figure S5. Distributions of Composite Construct Scores (N = 61)**

*Note.* Histograms of composite mean scores (arithmetic item means, 1–5 scale) for all five retained constructs. PLF Help Wanted (M = 3.91, SD = 0.70) and Expected PLF Impact (M = 3.78, SD = 0.70) were positively skewed toward higher values. PLF Cost Perception (M = 2.95, SD = 1.08) was approximately symmetric near the scale midpoint. PLF Awareness (M = 3.50, SD = 0.83) and PLF Subjective Norms (M = 3.11, SD = 0.95) showed moderate spread. Higher values indicate greater awareness, more positive evaluative beliefs, stronger perceived norms, or greater perceived client need for PLF-based assistance.


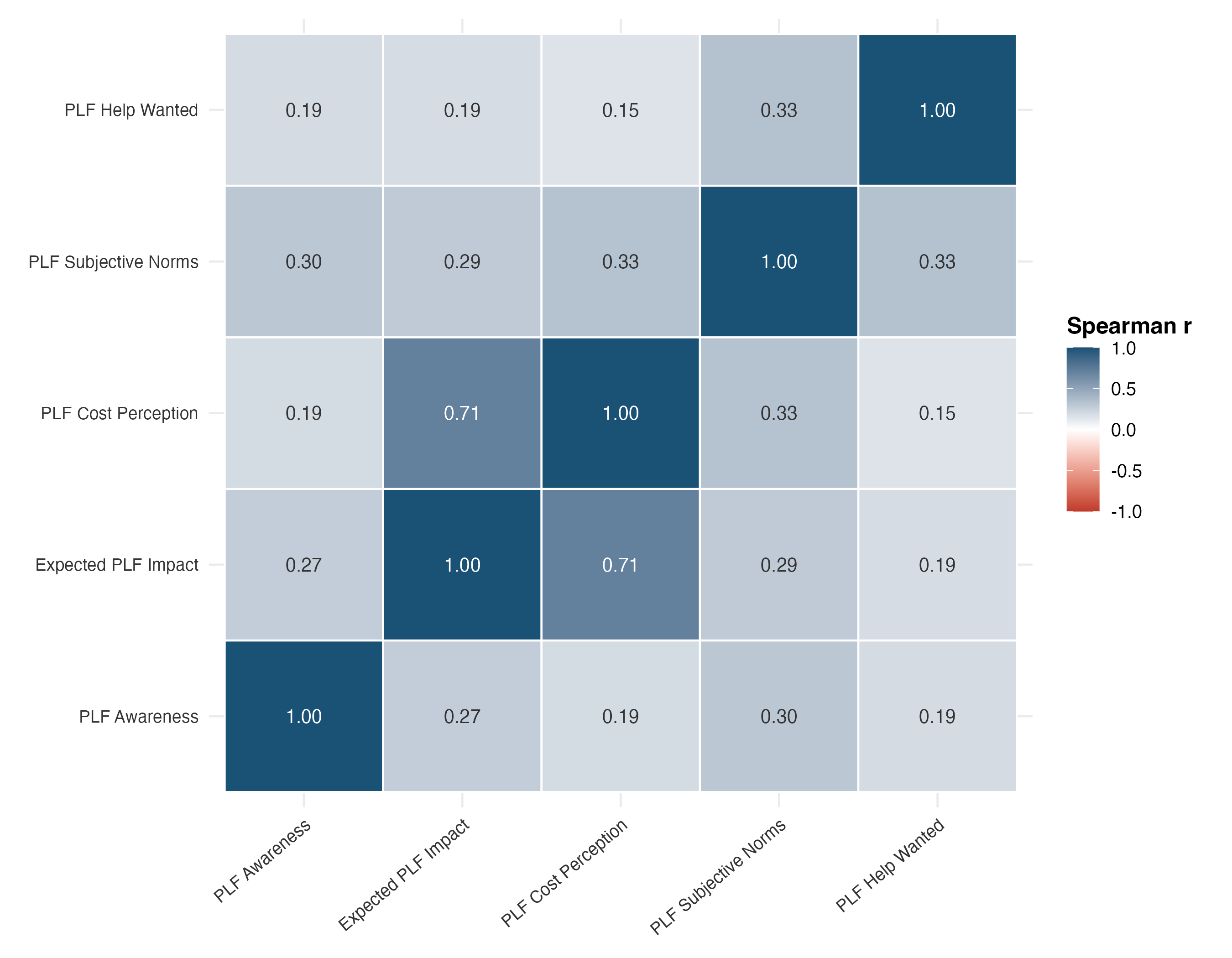


**Figure S6. Spearman Rank Correlation Heatmap — Five Construct Scores**

*Note.* Spearman rank correlations (ρ) among the five composite construct scores. Color intensity reflects correlation strength: dark blue = strong positive association; light blue/white = weak association; red = negative association (none observed here). The strongest pair-wise association was between Expected PLF Impact and PLF Cost Perception (ρ = 0.71, p < 0.001), indicating that veterinarians who anticipated greater PLF benefits also judged the financial investment as more justifiable. All other correlations were weak to moderate (ρ = 0.15–0.33).


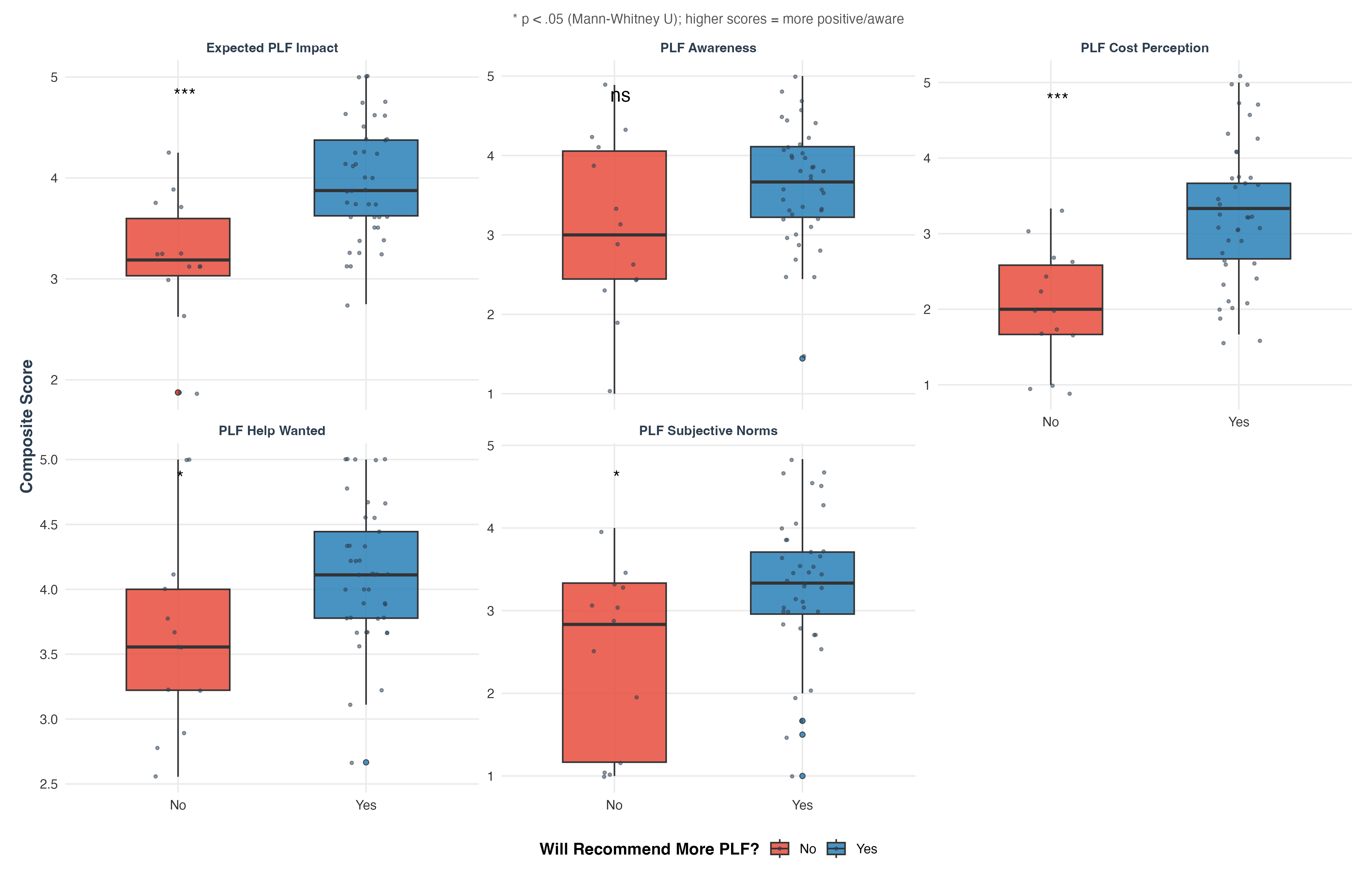


**Figure S7. Construct Scores by PLF Recommendation Intention Group (Mann–Whitney U)**

*Note.* Box plots of composite construct scores (1–5 scale) stratified by veterinarian PLF recommendation intention (No: n = 14; Yes: n = 41). Individual observations are overlaid as jittered points. Significance annotations from two-tailed Mann–Whitney U tests: *** p < 0.001, * p < 0.05, ns = not significant. Expected PLF Impact (W = 476.5, p < 0.001) and PLF Cost Perception (W = 486.0, p < 0.001) showed the largest group differences. PLF Awareness did not significantly differentiate the groups (W = 372.5, p = 0.100).


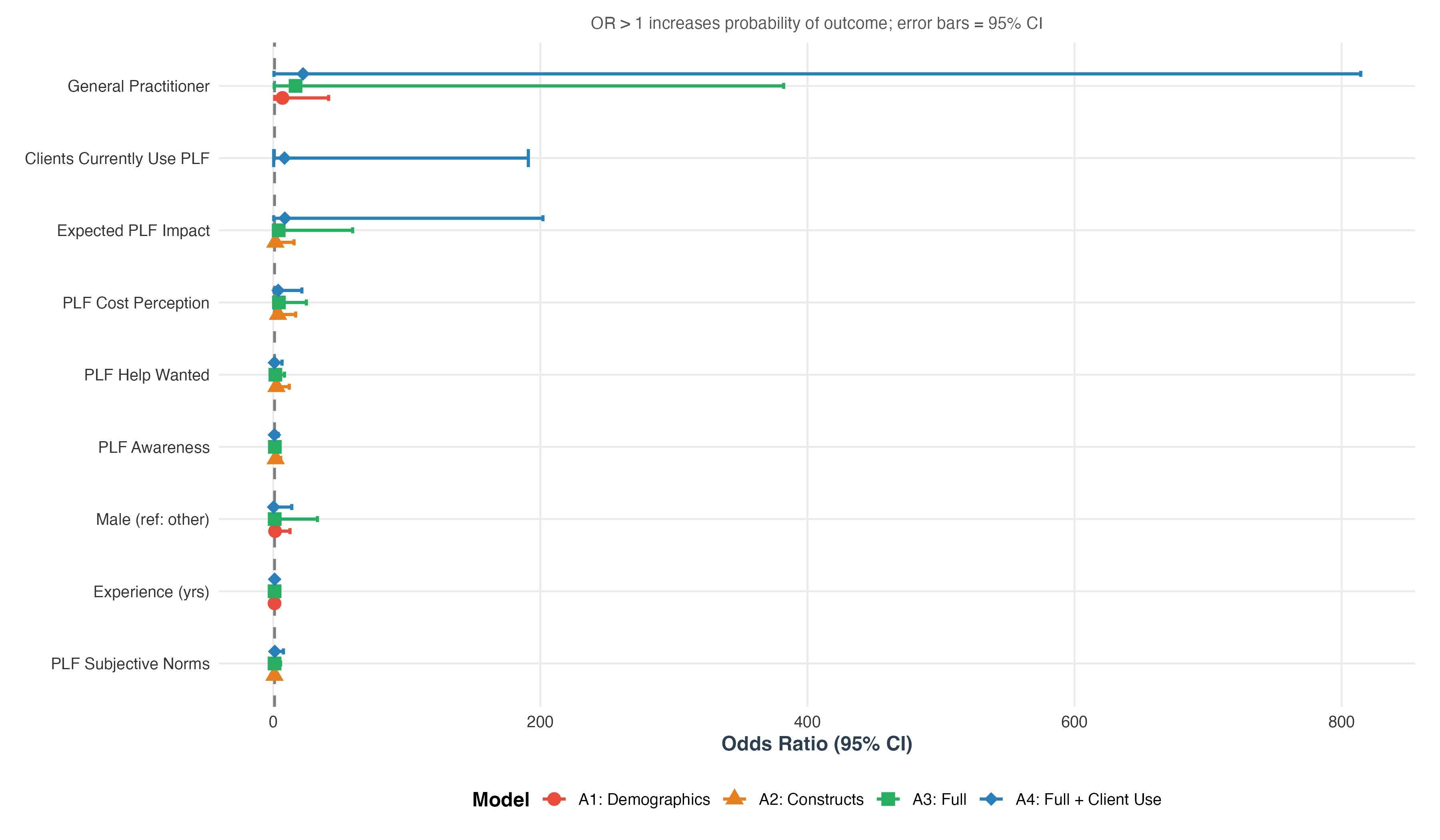


**Figure S8. Forest Plot of Odds Ratios — Outcome A (Veterinarian Intention to Recommend More PLF)**

*Note.* Odds ratios (OR) with 95% confidence intervals for predictors of veterinarian PLF recommendation intention across Models A1–A4 (n = 39 complete cases). OR > 1 indicates higher probability of intending to recommend more PLF. Error bars represent 95% CIs. Due to the small sample, CIs are wide and no individual TPB construct predictor reached conventional significance in the full model (Model A3). General Practitioner specialty was the only individually significant predictor in Model A1 (OR = 6.94, p = 0.034). Results are directional and exploratory.


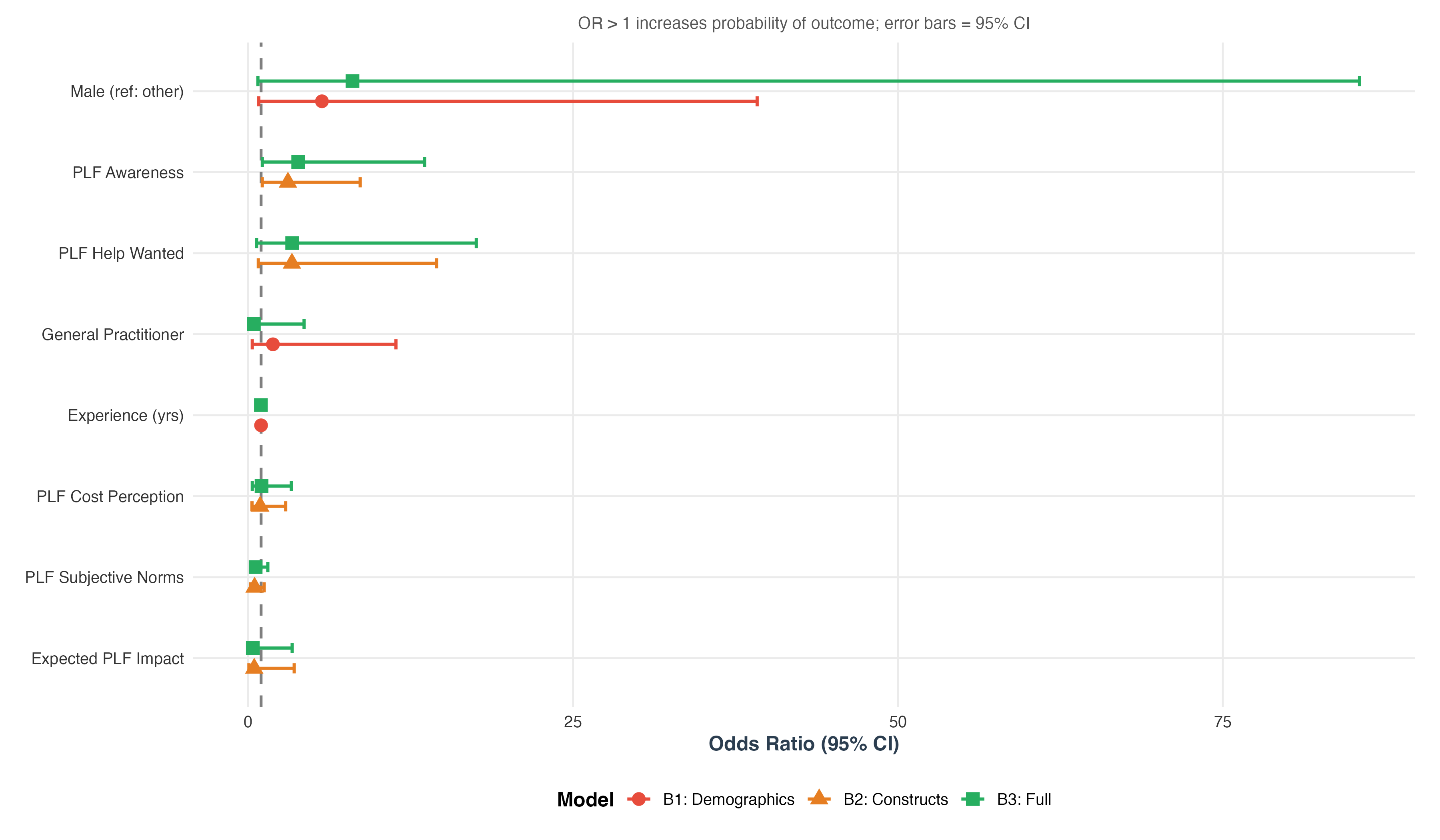


**Figure S9. Forest Plot of Odds Ratios — Outcome B (Clients Currently Using PLF)**

*Note.* Odds ratios (OR) with 95% confidence intervals for predictors of current client PLF adoption across Models B1–B3 (n = 39 complete cases). OR > 1 indicates higher probability that the veterinarian’s clients currently use PLF. Error bars represent 95% CIs. PLF Awareness was the only significant predictor across models (Model B2: OR = 3.07, p = 0.034; Model B3: OR = 3.85, p = 0.036). Results are directional and exploratory.


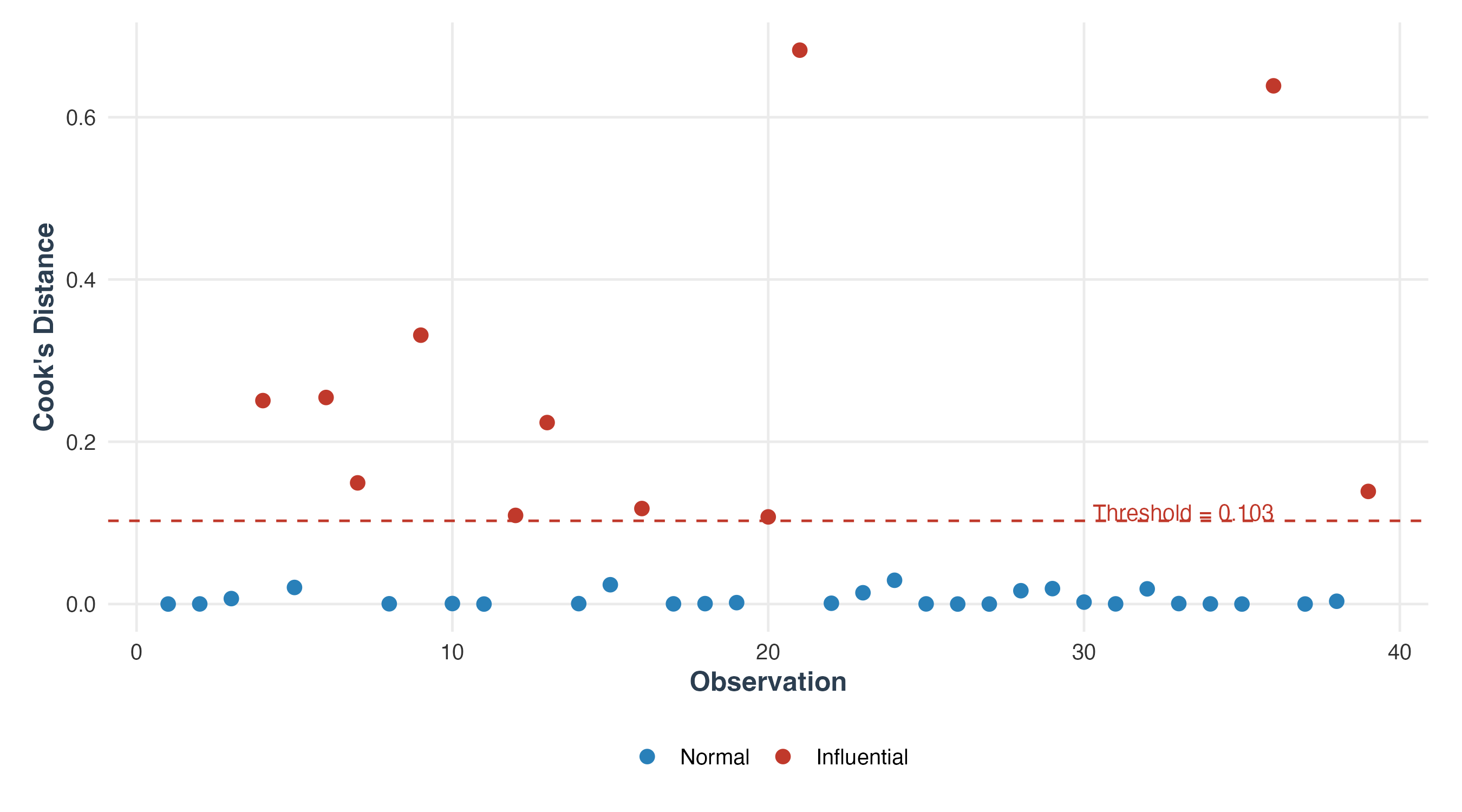


**Figure S10. Cook’s Distance Diagnostic Plot — Model A3 (Outcome A)**

*Note.* Cook’s distance values for each observation in the complete-case analytical sample (n = 39) for the full logistic regression model predicting veterinarian PLF recommendation intention (Model A3). The dashed red line marks the conventional influence threshold of 4/n = 0.103 (Cook, 1977). Red points (Influential) exceed this threshold. Removal of influential observations in sensitivity analyses did not materially alter the direction or pattern of coefficient estimates.


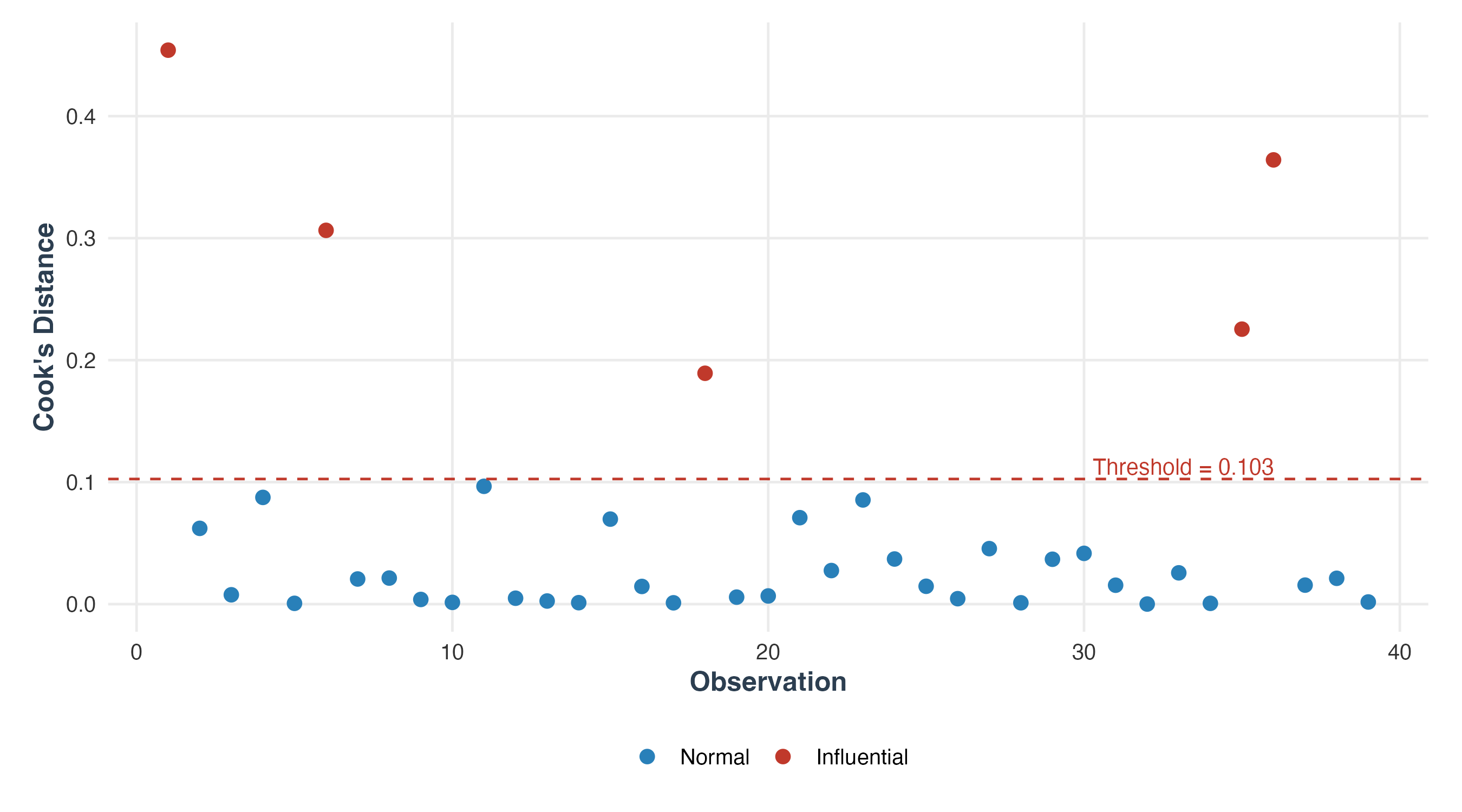


**Figure S11. Cook’s Distance Diagnostic Plot — Model B3 (Outcome B)**

*Note.* Cook’s distance values for each observation in the complete-case analytical sample (n = 39) for the full logistic regression model predicting current client PLF adoption (Model B3). The dashed red line marks the conventional influence threshold of 4/n = 0.103 (Cook, 1977). Red points (Influential) exceed this threshold. Removal of influential observations in sensitivity analyses did not materially alter the direction or pattern of coefficient estimates.
